# An overview of pharmacological effects of Crocus sativous and its constituents

**DOI:** 10.22038/IJBMS.2023.73410.15950

**Published:** 2024

**Authors:** Saeideh Saadat, Zahra Ghasemi, Arghavan Memarzia, Sepideh Behrouz, Mohammad Reza Aslani, Mohammad Hossein Boskabady

**Affiliations:** 1 Department of Physiology, School of Medicine, Zahedan University of Medical Sciences, Zahedan, Iran; 2 Applied Biomedical Research Center, Mashhad University of Medical Sciences, Mashhad, Iran; 3 Department of Physiology, Faculty of Medicine, Mashhad University of Medical Sciences, Mashhad, Iran; 4 Student Research Committee, Mashhad University of Medical Sciences, Mashhad, Iran; 5 Lung Diseases Research Center, Faculty of Medicine, Ardabil University of Medical Sciences, Ardabil, Iran; # These authors contributed equally to this work

**Keywords:** Crocetin, Crocin, Crocus sativus, Pharmacological action, Saffron, Safranal

## Abstract

*Crocus sativus* L. was used for the treatment of a wide range of disorders in traditional medicine. Due to the extensive protective and treatment properties of *C. sativus* and its constituents in various diseases, the purpose of this review is to collect a summary of its effects, on experimental studies, both *in vitro* and *in vivo*. Databases such as PubMed, Science Direct, and Scopus were explored until January 2023 by employing suitable keywords. Several investigations have indicated that the therapeutic properties of *C. sativus* may be due to its anti-oxidant and anti-inflammatory effects on the nervous, cardiovascular, immune, and respiratory systems. Further research has shown that its petals also have anticonvulsant properties. Pharmacological studies have shown that crocetin and safranal have anti-oxidant properties and through inhibiting the release of free radicals lead to the prevention of disorders such as tumor cell proliferation, atherosclerosis, hepatotoxicity, bladder toxicity, and ethanol induced hippocampal disorders. Numerous studies have been performed on the effect of *C. sativus* and its constituents in laboratory animal models under in vitro and in vivo conditions on various disorders. This is necessary but not enough and more clinical trials are needed to investigate unknown aspects of the therapeutic properties of *C. sativus* and its main constituents in different disorders.

## Introduction

The utilization of *Crocus sativus* L. dates back to a period of 3500 years. In the nation of Iran, the expanse dedicated to the cultivation of *C. sativus* amounts to approximately 1.64 million square kilometers (1, 2). *C. sativus* was extensively utilized in Asia as a potent medicinal herb in the management of coronary artery disease, hypertension, gastrointestinal ailments, irregularities in the menstrual cycle, and impairments in memory and cognitive function. Furthermore, it serves as a widely employed spice in the culinary industry. A multitude of investigations have indicated that the therapeutic attributes of *C. sativus* may be attributed to its anti-oxidant and anti-inflammatory effects on the nervous, cardiovascular, immune, and respiratory systems. Both animal and human studies on *C. sativus* extract have evidenced that this botanical specimen exhibits anticonvulsant and anti-Alzheimer properties (3, 4). For a long time in ancient Iran, Egypt, and Europe, *C. sativus* was used as a medicinal plant in the treatment of back pain, diabetes, and measles. Other uses of the plant include treatment for pre-eclampsia, abscesses, and wound healing. Today, modern research studies have shown that *C. sativus* compounds have healing properties, including anti-cancer, anti-diabetic, and analgesic activities. It also prevents renal ischemia and enlarged liver and spleen, as well as relaxing smooth muscles (5). 

Due to the extensive protective and therapeutic properties of *C. sativus* and its constituents in various diseases, the purpose of this review is to collect a summary of various pharmacological properties of *C. sativus* and its constituents, in both *in vitro* and *in vivo* experimental studies.

***Usage in traditional medicine***

The history of *C. sativus* cultivation dates back centuries and is now cultivated in countries such as Iran, India, Spain, Greece, and Turkiye. The demand for *C. sativus* cultivation has increased due to its widespread pharmacology leading to the widespread cultivation of *C. sativus* around the world (6). *C. sativus* cultivation has been increasing for many years due to its late fruiting as well as the difficulty of cultivating it, and its crop has even been abandoned in many countries of the world. At present, traditional cultivation methods have been replaced by mechanized and machine methods, and there are no restrictions on the collection of this crop (7, 8). 

In Iranian medical books, *C. sativus* is introduced as a hot and dry spice (9), and its properties are introduced with the words “*Moder”*, “*Moghavi”*, “*Mohalel”*, “*Molatef”*, and “*Monaghi”*, which mean tonic, resolvent, attenuant, and abstergent, respectively (10). Many studies have shown that *C. sativus* and its active constituents have extensive bioactivity and pharmacological properties. The traditional uses of *C. sativus* in ancient times are summarized in Table 1.

Today in the world, there is a strong tendency to use medicinal plants to treat diseases. This tendency is due to the high cost and side effects of industrial drugs on several illnesses and not exactly necessarily because herbal medicines are more effective on diseases (2). In addition to therapeutic uses, in ancient Iran, *C. sativus* with gold, flowers, and sweets was used in celebrations. It’s also the most important compound in the most powerful drugs of the past and has been used as an anti-inflammatory drug in cough, sore throat, cold swellings, otitis, and wounds (11). 

Between the 13th and 18th centuries, the main source of medical education in the West was the Qanoon Felteb and Kitab al-Hawi books, written by Avicenna and Rhazes, respectively (12). In ancient India, having a lot of *C. sativus* was a sign of wealth and royalty. Homemade remedies, herbal formulations, anti-poisonous, and Ayurvedic medicine were recommended for the applications of *C. sativus* (13). In Indian Ayurvedic medicine literature, *C. sativus* was introduced as an adaptogen. Other properties of this plant are cardiac tonic, nervine tonic, livotonic, diaphoretic, diuretic, carminative, emmenagogue, lactogogue, febrifuge, stimulant, relaxant, sedative, antistress, and anti-anxiety (14). *C. sativus* has also been used to revitalize facial skin, cleanse the liver of bile, treat cough, heal diaphragmitis, and as a substance to reduce eye inflammation in ancient Rome (15). 

The Romans also used *C. sativus* to treat jaundice and to clear bile (16). Historical documents show that *C. sativus* was first cultivated during the reign of the Media in parts of the Zagros and Alvand Mountains (17). Razi stated that the use of *C. sativus* due to euphoria leads to a psychotic state (18). *C. sativus* has traditionally been prescribed to maintain lung tone, improve respiratory function, and treat asthma. It also protects the cardiovascular system, improves cardiovascular function, and treats heart palpitations by maintaining heart tone. In addition to enhancing blood circulation and providing appropriate nourishment to the cardiac organ, *C. sativus* also exhibits antithrombotic and thrombolytic properties. This plant is known as a strong liver protector and prevents liver blockage in the Gastro-hepatic system. Tabari identifies properties such as hotness, moderateness, dryness, water solubility, and bitterness for *C. sativus* and believes that these properties can be effective in treating liver obstruction (19).

***Phytochemistry***

**Figure 1:**
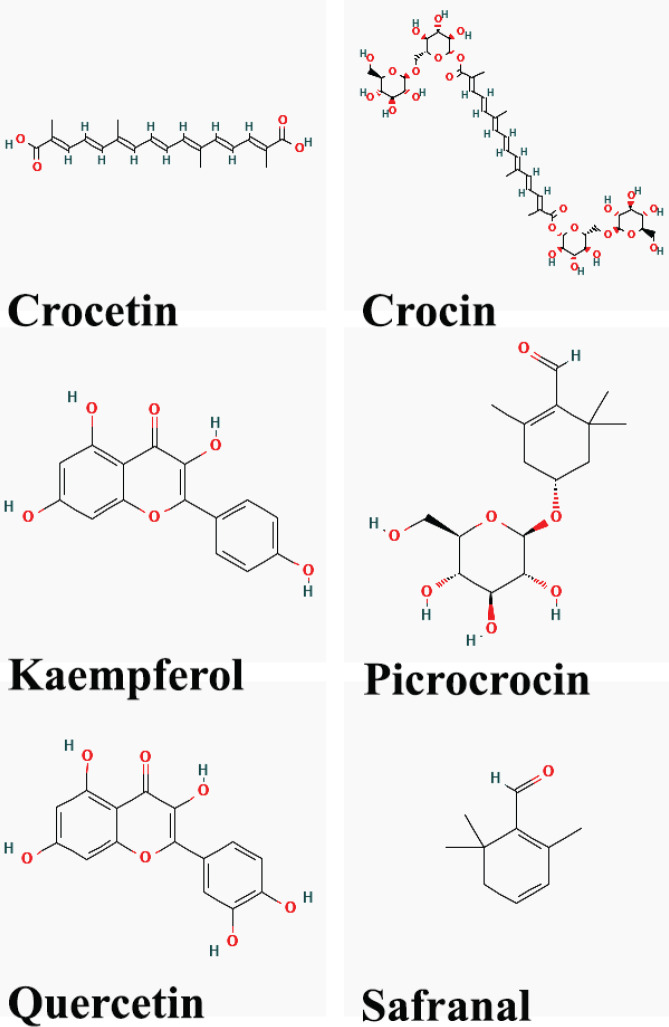
Chemical structure of chemical constituents of *Crocus sativus*

Over 150 compounds of both non-volatile and volatile nature have been identified in *C. sativus*. These compounds encompass proteins, amino acids, carbohydrates, minerals, vitamins, and pigments (20). The non-volatile constituents of the plant consist of crocins, crocetin, picrocrocin, quercetin, and kaempferol. On the other hand, the volatile compounds include terpenes and their esters, with safranal being the primary chemical component (21). The color of saffron, which serves as a natural food colorant, is attributed to crocins (C_44_H_64_O_24_, MW: 976.96), which are the glucosyl esters of crocetin (C_20_H_24_O_4_, MW: 328.4)(21). Safranal (C_10_H_14_O, MW: 150.21), a monoterpene aldehyde, is responsible for the characteristic aroma of *C. sativus* stigma (21). Picrocrocin (C_16_H_26_O_7_, MW: 330.37), a crystalline terpene-glucoside of safranal, causes actual taste of *C. sativus* and is the precursor of safranal (21). In addition, glycoside derivatives of quercetin (C_15_H_10_O_7_, MW: 302.236) and kaempferol (C_15_H_10_O_6_, MW: 286.23) are also the major flavonoid compounds in saffron petals (22, 23), (Table 2 and Figure 1).

***Method***

In this comprehensive review, the keywords including “*Crocus sativus*”, “Saffron”, “safranal”, “crocin” “crocetin”, “cancer”, “cardiovascular”, “gastrointestinal”, “renal” and “metabolic disorders” were searched in the popular search engines and databases including Iran Medex, Google Scholar, Medline, Pubmed, Scopus, and Wiley Online Library until the end of January 2023 to identify articles that explain numerous experimental effects of *C. sativus* and its main constituents on various diseases.

## Results

***Anti-cancer effect***

*C. sativus*

The ethanolic extract derived from the plant *C. sativus* has the potential to exert a fatal impact on human hepatocellular carcinoma cells (HepG2) as well as human cervical carcinoma cells (HeLa), primarily through the induction of apoptosis. This extract effectively eliminates tumor cells without causing any adverse effects on normal cells (24). The aqueous extract of *C. sativus* demonstrates the capability to induce intoxication in both hepatocellular carcinoma (hepg-2) and laryngeal carcinoma (Hep-2) cell lines through its ability to restrict the production of nitric oxide (NO)(25).

Treatment with *C. sativus* in human pancreatic cancer cell lines (bxpc-3) and other cancer cells resulted in the initiation of apoptosis via G1-phase cell cycle arrest of bxpc-3 cells and consequently diminished tumor progression (26). 

The administration of an aqueous extract derived from* C. sativus *to both human transitional cell carcinoma (TCC) and mouse non-neoplastic fibroblast cell lines demonstrated a notable suppression of cellular division and proliferation (27). 

Consuming an aqueous extract of *C. sativus* (100 to 800 μg/ml) in human breast carcinoma cells showed inhibitory effects on matrix metalloproteinase gene expression dose-dependently which was highest at its concentration of 200 μg/ml (28).

The extract obtained from Zhejiang *C. sativus* has exhibited notable anti-proliferative and pro-apoptotic properties. This has been observed through the modulation of cell proliferation activity and induction of apoptosis in human non-small cell (A549) and small cell lung cancer cell lines (H446) *in vivo*. Moreover, the administration of *C. sativus* extract (at a dosage of 100 mg/kg, orally for a duration of 28 days) has been found to induce cell apoptosis, resulting in a reduction in xenograft tumor size. This effect is attributed to the activation of caspase-3, -8, and -9 pathways (29).

Treatment using an extract derived from *C. sativus*, as well as the compounds crocin and crocetin, exhibited the ability to reduce tumor growth in male mice afflicted with prostate cancer, specifically the PC3 and 22rv1 cell lines. This reduction was achieved through the down-regulation of N-cadherin and beta-catenin, coupled with an increase in E-cadherin expression. Consequently, this process effectively suppressed the occurrence of epithelial-mesenchymal transition (EMT). Moreover, the inhibition of prostate cancer cell invasion and migration was attributed to the decreased expression and activity of metalloproteinase and urokinase. Remarkably, the antitumor effects of crocetin surpassed those exhibited by the other two compounds (30). 

*C. sativus* can be used as an anti-cancer agent in two ways, inhibition of the cell cycle by targeting the DNA sequence and modulating gene expression, which leads to cessation of cell proliferation in the early stages, and activation of apoptosis, which leads to the death of cancer cells. In diethyl nitrosamine (DEN)-induced liver cancer in rats, *C. sativus* treatment through these two methods led to chemopreventive action against liver cancer cells. DEN led to the formation of structures called hepatic dyschromatic nodules in the liver tissue but in contrast, *C. sativus* reduced it. Based on these observations, *C. sativus* has hepatoprotective effects in liver cancer through the induction of apoptosis, inhibition of cell proliferation, and inflammatory and anti-oxidant activities (31).

Topical use of aqueous extract of *C. sativus* (100 mg/kg) reduced skin carcinogenesis, and methylchloanthrene (MCA) induced soft tissue sarcomas in mice through inhibition of apoptosis induction (32). In addition, the plant modulated inflammatory response and inhibited oxidative damage in DEN-induced hepatic cancer (31).

The formation of papillomas in female Swiss albino mice with skin carcinogenesis induced by dimethyl benz[a] anthracin (DMBA) was hindered by the administration of an aqueous infusion of *C. sativus* (50-500 mg/kg). This hindrance was achieved by altering the activity of phase II detoxifying enzymes, namely glutathione peroxidase (GPX), glutathione-S-transferase (GST), catalase (CAT), and superoxide dismutase (SOD) (33).

Consuming an aqueous extract of *C. sativus* as a daily supplement in the kidney cancer model led to a reduction in the oxidative effects of chemotherapy due to cisplatin, and reduced renal excretion (34). In cisplatin-, mitomycin-C- and urethane-induced mice chromosomal damage, pretreatment with dried stigmas of *C. sativus* (25, 50, and 100 mg/kg), spatially high doses of *C. sativus*, significantly reduced the genotoxicity of this genotoxin. However, all three doses showed a protective effect against urethane (35). Pre-treatment with aqueous extract of *C. sativus* reduced the side effects of drugs genotoxicity (cisplatin, urethane, cyclophosphamide, and mitomycin-C) and decreased their oxidative effects. Therefore, it can play a moderating role in peroxidation and detoxification caused by chemotherapy (36).

*Crocin*

The inhibitory properties of crocin have been demonstrated through the utilization of the MTS assay in three distinct cell lines, namely HCT-116, SW-480, and HT-29. It is important to note that these effects were observed exclusively in cancerous cells, as non-cancerous cells remained unaffected (37). Crocin increased the Bax/Bcl-2 ratio to induce apoptosis, and tumor proliferation and growth (38). Administration of 250 and 500 μg/kg crocin after melanoma lung metastasis implantation, decreased uronic acid, hexosamine, hydroxyproline, gamma-glutamyl transpeptidase (g-GGT), and serum sialic acid, which were metastasis-induced biomarkers, and crocin prevented the expression of genes such as vascular endothelial growth factor (VEGF), ERK-2, matrix metalloproteinase (MMP)-2, MMP-9, and K-ras (39).

In the experimental model of colorectal cancer in animals induced by rat adenocarcinoma DHD/K12-prob cells, prolonged administration of crocin resulted in a reduction in tumor growth and an increase in survival time (40). Crocin was subjected to testing on both animal and human colon adenocarcinoma cells (DHD/K12-prob and HT-29), demonstrating a significant cytotoxic effect on these cells, leading to the eradication of cancer cells and a decrease in tumor growth (40). Moreover, the utilization of pegylated nanoliposomes containing crocin resulted in cytotoxicity against colon carcinoma (C-26) cells in laboratory settings (41). 

Crocin was found to induce inhibition of the cell cycle progression and apoptosis in breast cancer tumors through the down-regulation of cyclin D1 and p21Cip1 expression (42). The combined administration of crocin and crocetin in mice with breast cancer led to a reduction in the growth of cancerous tumors, except that crocin had a greater protective effect in the early stages of tumor growth (43). 

*Crocetin*

Crocetin, trans-crocin-4, and safranal exhibit remarkable efficacy in impeding the proliferation of breast cancer cell tumor lines, namely MDAMB-231 and MCF-7, whilst simultaneously manifesting anti-proliferative attributes against breast cancer cells (44). The co-administration of crocin alongside gamma radiation or paclitaxel therapy instigated apoptosis and engendered a synergistic impact on reducing the survival rate in MCF-7 breast cancer cells (45). 

In both *in vivo* (50, 100, 200 µM/L) and *in vitro* (4 mg/kg, for 30 days) studies, crocetin exhibited inhibitory effects on cell proliferation in pancreatic cancer cells by significantly modifying the expression of Cdc-2, Cdc-25C, Cyclin-B1, and epidermal growth factor receptor. Moreover, crocetin also reduced the proliferation process and H3-thymidine incorporation in various cancer cells such as BxPC-3, Capan-1, and ASPC-1. Additionally, it induced apoptosis through the modulation of the Bax/Bcl-2 ratio (38). Crocetin effectively suppressed the proliferation and invasiveness of highly invasive breast cancer cells by down-regulating the expression of matrix metalloproteinases in MDA-MB-231 cells (46). 

Injection of crocetin in a lung cancer model induced by benzopyrene in Swiss albino mice resulted in a reduction of cell proliferation by 45% and 68% after 8 and 18 weeks of treatment, respectively. The crocetin compound exhibited both preventive and therapeutic effects on benzopyrene-induced lung cancer in the animal model, demonstrating its potential as an anticarcinogenic agent. These effects were attributed to the inhibitory impact of crocetin on polyamine synthesis and alterations in glycoprotein levels in lung cancer (47). Furthermore, crocetin demonstrated the ability to suppress cell proliferation by inhibiting glycoprotein and polyamine synthesis, thereby affecting proliferating cells (47).

Administration of crocetin (20 mg/kg) as a pretreatment before and after induction of lung cancer by Benzo(a) pyrene B(a)p (50 mg/kg, orally) in mice showed its anti-tumor activity through increasing activity of anti-oxidants and glutathione metabolizing enzymes in both liver and lung mice tissue (48). Treatment with crocetin after the initiation of colitis by 2, 4, 6-trinitrobenzene sulfonic acid (TNBS) resulted in a reduction in the levels of malondialdehyde (MDA), the expression of TH1 and TH2 cytokines, and inducible NO synthase due to the down-regulation of nuclear factor kappa B (NF-κB). These alterations consequently hindered the occurrence of colorectal cancer induced by colitis using the regulation of specific proteins (49).

Crocetin decreased the concentration of hepatic enzymes including aspartate aminotransferase (AST), alanine aminotransferase (ALT), alkaline phosphatase (ALP), as well as gamma-glutamyl transpeptidase, while effectively managing hepatotoxic lesions instigated by aflatoxin B1 (AFB1) (50). Administration of crocetin supplementation (10, 20, and 40 mg/kg) reduced the levels of oxidants such as MDA, IL-1𝛽, tumor necrosis factor-alpha (TNF-α) levels and the number of polymorphonuclear cells (PMN) in mice with uterine cervical cancer induced by methylcholanthrene (MCA)(51).

*Safranal*

Safranal can be considered an anti-cancer agent by preventing gene toxicity. This substance can protect against DNA damage caused by Methyl methane sulfonate (MMS)(52). Table 3 presents a summary of the anti-cancer properties exhibited by *C. sativus* and its constituents.

***Cardiovascular diseases***

*Crocus sativus*

Numerous studies have proven the anti-oxidant effects of *C. sativus* which alleviate ischemia-reperfusion (IR) injuries of the heart. Left ventricle end-diastolic pressure (LVEDP), coronary flow, heart rate, and left ventricle pressure were improved 6 months after oral consumption of *C. sativus*. In addition, *C. sativus* activated the GPX, decreased lipid peroxidation, induced restoration of the phosphorylation level of Akt and 4EBP1, and reduced the activity of p38 and infarct size (53). Aqueous-ethanolic extract of *C. sativus* blocked calcium channels in the isolated myocardium of guinea pig heart and decreased myocardial contractility (54). *C. sativus *ethanolic extract reduced heart rate and contractility through Ca^2+^ channel blockage in guinea pigs (54). Oral administration of *C. sativus* extract (100 mg/kg) as a pretreatment through electrical conductivity reduction and prolonging action potential period, prevented the occurrence of lethal ventricular arrhythmia caused by I/R injury (55).

The application of hydroalcoholic extract derived from *C. sativus* at a dosage of 200 mg/kg to hypertensive rats induced with NG-nitro-L-arginine methyl ester (L-NAME) yielded a reduction in cross-section area, media thickness, and elastic lamellae number. Additionally, a decrease in hypertension was observed (56). A recent investigation has demonstrated that the introduction of *C. sativus* extract, achieved using stimulation and subsequent production of NO, results in the fortification of the atrioventricular node’s (AV node) protective function against supraventricular arrhythmia in rabbits (57). Administration of an aqueous extract of *C. sativus* at varying doses (10, 20, and 40 mg/kg, IP) over a period of 5 weeks resulted in a reduction of mean systolic blood pressure (MSBP) in a dose-dependent manner in acid desoxycorticosterone acetate (DOCA)-induced hypertensive rats (58).

Intravenous (IV) injection of aqueous extract of *C. sativus *stigma (2.5, 5, and 10 mg/kg), crocin, and safranal reduced hypertension in normotensive and hypertensive anesthetized rats without activating tachycardia reﬂex. Although all three compounds improved heart function and reduced vasoconstriction, safranal had a stronger hypotensive effect on lowering blood pressure than the other two compounds. In contrast, in anesthetized rats, crocin had a stronger antihypertensive effect (59). 

*Crocin*

Intraperitoneal (IP) daily injection of crocin for three weeks improved arrhythmia which is followed by reperfusion. Heart IR led to a decrease in anti-oxidant agents such as SOD and glutathione (GSH) activities and an increase in MDA levels in the heart muscle. Treatment with crocin increased CAT activity by modulating all of these factors (60). Oral consumption of crocin (40 mg/kg) for 21 days showed the same effects as vitamin E, against oxidative injury in the treatment of cardiac I/R injury (61). Administration of crocin to three animal groups with 10, 20, and 40 mg/kg doses, showed cardiac protective effects against post-I/R after infarction. In the same study, co-administration of the highest dose of crocin (40 mg/kg) with vitamin E (100 mg/kg) reduced the size of myocardial infarction and improved the dynamic parameters (61). In bovine aortic endothelial cells, crocin increased the bcl‐2/bax ratio and expression and inhibited aortic endothelial cell apoptosis and atherosclerosis (62).

In hypertensive rats induced by DOCA-salt, the administration of crocin (50, 100, and 200 mg/kg, IP) over a period of 5 weeks resulted in a dose-dependent reduction in mean systolic blood pressure (MSBP) (58). In addition, crocin reversed the systolic blood pressure and heart rate in diazinon (DZN)-induced hypotension in rats (63). In animal models of hemorrhagic shock, IV injection of crocin (60 mg/kg) in the initial stages of resuscitation in mice, increased arterial PO2 and decreased PCO2. This action led to a reduction in MDA, TNF-α, and IL-6 serum levels and increased IL-10. It also prevented NF-kb pathway activation. These processes ultimately protect the lungs from tissue damage caused by ischemia (64).

In a separate investigation employing an identical protocol, the administration of crocin (at a dosage of 60 mg/kg) was intravenously introduced into the animal subsequent to the induction of hemorrhagic shock through blood withdrawal. The findings indicated a reduction in tissue damage in various organs, including the kidney, liver, pancreas, and muscle, as a result of crocin administration. This phenomenon occurs using the restriction of the activation of the NF-κB pathway in lung tissue, the inhibition of serum concentrations of the proinflammatory cytokines TNF-α and IL-6, and an elevation in the level of the anti-inflammatory cytokine IL-10. Collectively, these effects have the potential to mitigate the deleterious consequences associated with the release of tissue inflammatory factors during episodes of hemorrhagic shock (64).

*Crocetin*

In the context of atherosclerosis in rats induced by a high cholesterol diet (HCD), the administration of crocetin at dosages ranging from 25 to 50 mg/kg over 10 weeks exhibited beneficial effects on the lipid profile as well as other inflammatory mediators. Crocetin also augmented lipid profile toward standard value, and co-administration of crocetin with simvastatin certificated dyslipidemia, through increased anti-oxidant activity and inhibition of phosphorylated p38 mitogen-activated protein kinase (MAPK) (65). Crocetin inhibited the proliferation of vascular smooth muscle cells (VSMs) exposed to the angiotensin enzymes (ages) and also reduced the levels of inflammatory factors such as TNF-α and IL-6, and structural enzymes such as MMP-2 and MMP-9. These effects led to cardiovascular protection against the complications of diabetes (66).

Angiotensin II-induced VSM cell proliferation through activated extracellular signal-regulated kinases 1/2 (ERK1/2) and increased intracellular calcium (Ca2) concentration and extracellular Ca2 influx were inhibited by pretreatment with crocetin via Ca^2+^-dependent pathway blocking (67). Pre-incubated bovine endothelial cells (BEC) with crocetin (0.01, 0.1, and 1 μM) after exposure to advanced glycosylation end products (AGEs) suppressed the ICAM-1 protein and MMP up-regulation, reduced leukocyte adhesion and protected the mitochondrial function. It also increased anti-oxidant defense by decrease in MDA and O2^-^ levels and increase in SOD activity (68). Crocetin at a concentration of 1 μM was found to impede the migration of vascular smooth muscle cells (VSMCs) induced by advanced glycation end products (AGEs). This inhibition was achieved through the down-regulation of tumor necrosis factor-alpha (TNF-α), interleukin-6 (IL-6), matrix metalloproteinase-2 (MMP-2), and matrix metalloproteinase-9 (MMP-9) (66).

Vascular permeability in fibroblasts and human umbilical vein endothelial cells (HUVECs) is controlled by cadherin which is a key protein that controls the permeability of these cells. Crocetin treatment increased vascular endothelial-cadherin expression in tissues and suppressed cellular inflammatory infiltration (69). Crocetin reduced VEGF-induced tube formation by HUVECs and migration of HRMECs, p38 and protected VE-cadherin (70).

In an experimental rat model of IR, administration of crocetin (at a dose of 50 mg/kg) resulted in a decrease in cardiac damage, oxidative stress, and inflammation. This was evidenced by a reduction in the size of the infarct, levels of creatine kinase-MB (CK-MB), MDA, and TNF-α, as well as an increase in the activity of total SOD and the anti-inflammatory cytokine IL-10 (71). 

Crocetin (25 and 50 mg/kg, IP, for 15 days) treatment on norepinephrine-induced cardiac hypertrophy, significantly improved myocardial function compared to captopril as a standard drug through increased SOD and GPX activities and decreased lipid peroxidation in the cardiac myocytes. However, captopril showed a stronger effect on left ventricular index improvement (72). The administration of crocetin demonstrated an augmentation in the functionality of voltage-dependent pumps, including Na/K-ATPase in cardiac cells and Ca2/Mg2-ATPase in mitochondria. Additionally, there was a notable decrease observed in the expression of MMP-2 and MMP-9 mRNA. Crocetin inhibited energy metabolism disruption in noradrenaline-induced apoptosis in cardiac myocytes through increased mitochondrial membrane potential due to activation of Na/K ATPase and Ca ATPase pumps as well as induction of mitochondrial succinic dehydrogenase activity (73).

Administration of crocetin (50 mg/kg, thrice daily over a duration of one week) to adult male C57/B6 mice with cardiac hypertrophy induced by aortic banding (AB) not only improved hypertrophy but also reversed the course of the injury. The observed impacts were a result of the inhibition of the reactive oxygen species (ROS)-dependent MAPK/extracellular signal-regulated kinase-1/2 (ERK1/2) pathway and stimulation of GATA binding protein 4 (GATA-4) activation. At the molecular level, crocetin inhibited hypertrophy by blocking NF-κB signaling (74).

Treatment of LDL-induced atherosclerosis in rabbits with crocetin in both *in vivo* and *in vitro* conditions increased endothelial NO synthase (eNOS) activity in aortic endothelial cells due to enhancement of NO production. This compound is also capable of relaxing the thoracic aorta (75). One of the major growth factors that plays a key role in the proliferation of VSMCs is angiotensin II (Ang II), which is produced as a result of the activation of the renin-angiotensin system. The studies indicated that crocetin suppresses Ang II-induced VSMC proliferation via inhibited phosphorylation, activation, and nuclear translocation of extracellular signal-regulated kinase1/2 (ERK1/2) (76). 

Treatment with crocetin on bovine aortic VSMCs induced by Ang II effectively obstructed the progression of the cell cycle initiated by Ang II, thus halting the cells in the G0/G1 phase. This modification hindered the activation of extracellular signal-regulated kinase1/2 (ERK1/2) and the subsequent expression of its downstream effector c-fos, which were initially stimulated by Ang II. Furthermore, it enhanced the activity of SOD and led to a decrease in intracellular ROS (69).

*Safranal*

Administration of Crocin (50, 100, and 200 mg/kg, IV), safranal (0.25, 0.5, and 1 mg/kg) and the aqueous extract of *C. Sativus* (2.5, 5, and 10 mg/kg) to normotensive and hypertensive anesthetized rats induced by DOCA, decreased mean arterial blood pressure (MABP) in both of them (77). In another study, administration of safranal (1, 2, and 4 mg/kg, IP) for 5 weeks reduced the MSBP in a dose-dependent manner (58).

Safranal treatment (0.1-0.5 ml/kg/day, IP) for two weeks in IR-heart induced by occluded left anterior descending coronary artery, resulted in enhanced left ventricular functionalities and decreased infarct size, plausibly using Akt/GSK-3b (glycogen synthase kinase)/eNOS pathway phosphorylation and the suppression of IKK-b/nfқb protein expression in cardiac cells. In addition, safranal modulated the cardiac injury indicators such as lactate dehydrogenase (LDH) and CK-MB as well as reducing TNF-α levels, inflammatory cells, edema, and enhanced hemodynamic heart parameters. Therefore, safranal preserves the myocardial architecture of the heart (78). Table 4 presents a summary of the cardiovascular effects exhibited by *C. sativus* and its constituents.

***Central nervous system (CNS) disorders***

**Anti-depressant effect**

*C. sativus*

Administration of aqueous (160 and 320 mg/kg) and alcoholic (200 and 800 mg) extracts of *C. sativus* and its constituents improved depressive symptoms in depressed mice and showed better results than fluoxetine. In addition, the results showed more therapeutic potency and inactive time reduction compared to the control group (79). The antidepressant effect, resulting from an augmentation in climbing time and stereotypic activity, was likewise observed following the administration of aqueous and ethanolic extracts of *C. sativus* in mice (80). Treatment with the petal and ethanolic extracts of *C. sativus,* crocin, and safranal led to treatment of depression in mice (81). Clinical trials confirmed that *C. sativus* petals similar to fluoxetine can treat mild to moderate depression (82, 83).

*Crocin and Safranal*

Administration of crocin (50-600 ml/kg) and safranal (0.5 mg/kg) showed antidepressant activity using a forced swimming test in depressed mice (79). Table 5 presents a summary of the neuroprotective effects exhibited by *C. sativus* and its constituents.

**Anti-seizure effect**

*C. sativus*

In pentylenetetrazole (PTZ)-induced seizure animals models, the aqueous (0.08, 0.32, 0.56 and 0.80 g/kg, IP) and ethanolic (0.2-2.0 g/kg, IP) extracts of *C. sativus* reduced convulsant activity, onset of tonic convulsions, period of seizure using maximal electroshock seizure (MES) tests, and mortality in epileptic mice. At the dosage of 80 mg/kg, the aqueous extract exhibited a comparable efficacy to that of 10 mg/kg of phenobarbital in the animal models of PTZ-induced seizures (84). Both the aqueous (200, 400, and 800 mg/kg, IP) as well as the ethanolic (250 and 500 mg/kg, IP) extracts of *C. sativus* also yielded a noteworthy decrease in convulsion activity in rats with PTZ-induced seizures (85).

*Crocin*

Treatment of PTZ-kindled mice with crocin (5, 10, and 20 mg/kg, PO) reversed learning and memory impairments through protecting hippocampal pyramidal layer neurons (86). 

*Safranal*

Safranal (0.15 and 0.35 ml/kg, IP) reduced the duration of seizures and could be a protective agent against animal death in PTZ-induced convulsions in mice (87). Safranal (72.75, 145.5, and 291 mg/kg) exhibited a diminishing effect on the occurrence of minimal clonic and generalized tonic-clonic seizures in PTZ-induced seizure mice. This effect was found to be reliant on the dosage administered and occurred through the mechanism of interaction with the GABAA-benzodiazepine receptor (88). A single dose of safranal (291mg/kg, IP) showed an antiepileptic effect in acute epileptic laboratory animal models via modulating GABA receptors (89). Administration of safranal showed antiabsence seizure activity in epileptic C57BL/6 mice via modulating benzodiazepine binding sites of the GABAA receptor complex (89). Table 5 presents a summary of the neuroprotective effects exhibited by *C. sativus* and its constituents.

**Neuroprotective effects**

*C. sativus*

In the context of experimental autoimmune encephalomyelitis (EAE) mice, administration of an ethanolic extract derived from *C. sativus* led to a noteworthy mitigation of leukocyte infiltration within the spinal cord. Furthermore, this treatment exhibited a decline in clinical manifestations associated with EAE in C57BL/6 mice (90). In the context of rat experiments involving middle cerebral artery occlusion (MCAO), the administration of *C. sativus* extract prior to the onset of cerebral ischemia exhibited a significant impact. This impact was observed through the modulation of cerebral MDA, GPX, SOD, and CAT, as well as the regulation of glutamate and aspartate levels and the activity of Na+/K+ ATPase. These findings serve to demonstrate the protective properties of *C. sativus* stigma aqueous extract (100 mg/kg, PO) by means of inhibiting peroxidation and reducing glutathione levels and other anti-oxidant agents. The ultimate goal of these actions is to prevent neuronal cell death resulting from ischemic conditions (91). In neurodegenerative disorders such as Alzheimer’s and Parkinson’s, the extracts derived from the stigmas of *C. sativus* effectively impeded the formation of amyloid fibrils, displaying a concentration- and time-dependent manner (92). 

*C. sativus *extract (60 mg/kg) inhibited the memory loss processes in streptozocin (STZ)-induced Alzheimer rats (93). Administration of ethanolic extract of *C. sativus* and crocin in Alzheimer patients resulted in healing of Aβ brain pathology and decreased neuro-inflammation through increased blood-brain barrier of amyloid-β and apolipoprotein E (apoE), as well as degradation of related enzymes (94). Ethanolic extract of *C. sativus *(125 and 250 mg/kg, PO) on hippocampus dentate gyrus in anesthetized rats, reduced the ethanol-induced long-term potentiation (LTP)‐blockade and in higher dose (500 mg/kg, PO) reversed ethanol‐induced impairment in brain direct ethanol injection. The ethanolic extract also improved synaptic plasticity in the hippocampus through direct action on the CNS and peripheral function (95).

Neuropathic pain in the experimental animal model was attenuated by aqueous and ethanolic extracts of *C. sativus* (50, 100, and 200 mg/kg) and safranal (0.025, 0.05, and 0.1 mg/kg), and behavioral symptoms were improved (96). Suppression of oxidative stress, modulation of proinflammatory cytokines, and apoptosis attenuation are also the results of administration of aqueous and ethanolic extracts of *C. sativus* (200 mg/kg, IP)(97). Using passive avoidance tasks and object recognition, it was shown that *C. sativus* extract (SE at 30 and 60 mg/kg) can store and retrieve information and reduce side effects of scopolamine administration such as spatial memory disorder. Administration of the plant extract with crocins (30 mg/kg, and to a lesser extent, 15 mg/kg) showed similar results (98). Using the passive avoidance paradigm through the Y maze task, it was demonstrated that the aqueous extract of *C. sativus* (60 mg/kg, IP) as well as safranal (60 mg/kg, IP, for 3 weeks) exhibited the ability to restore cognitive abilities by enhancing learning and memory, while also counteracting the decline in cognitive function observed in rats.

Hydro-alcoholic extract of *C. sativus *(30 mg/kg, IP for 15 days) in amnestic mice induced by D-galactose and sodium nitrite (nano2), showed preventive and therapeutic effects in retrieval learning and memory in one-way passive and active avoidance tests (99). *C. sativus *hydro-ethanolic extract inhibited ketamine-induced behavioral defects by reducing extracellular glutamate levels. It also binds to NMDA receptors and inhibits the transfer of glutamate in synaptic apace in a concentration-dependent manner (100). *C. sativus* aqueous extract (150 and 450 mg/kg, IP, for 5 days, three days before and two days after the training phase) improved the time latency for entering the dark compartment in morphine-induced memory impairment male mice (101). *C. sativus* extract (10, 30, and 50 mg/kg, IP, for 5 consecutive days) also ameliorated spatial learning and memory in ethanol-induced mice and reversed the ethanol-induced hippocampal long-term weakening, and reduced side effects of morphine in a dose-dependent manner (102).

One of the notable consequences of the *C. sativus* extract on cognitive function is its efficacy in impeding the induction of hippocampal long-term potentiation (LTP) caused by ethanol. Administering *C. sativus* at a dosage of 250 mg/kg orally may prove to be efficacious in preventing the inhibition of LTP in the dentate gyrus caused by acetaldehyde (103). Using object recognition and the step-through passive avoidance task, it was shown that the extracts of *C. sativus* (30 and 60 g/kg, orally, 24 hr), improved memory through modulating storage and/or retrieval of information (104). *C. sativus* extract (1 mg/kg/day, IP) showed a protective effect against energy metabolism disorder in the 3-nitropropionic acid-induced mitochondrial toxicity (105). 

Administration of *C. sativus* significantly improved the memory and learning of adult and aged mice in the passive avoidance paradigm by reducing anti-oxidant factors and the caspase-3 enzyme activity modulation. *C. sativus* (1-250 µg/ml), crocetin, and safranal (1-125 µm) reduced the toxicity induced by hydrogen peroxide in neuroblastoma SH-SY5Y cells *in vitro* (106). *C. sativus* extract and its constituents, crocetin, dimethylcrocetin, and safranal bound to the docking site of acetylcholinesterase and increased acetylcholine levels in the synaptic space *in vitro* (107).

*C. sativus* aqueous extract (200 mg/kg), as well as honey syrup, demonstrated an ability to counteract the neurodegenerative damage induced by aluminum. This was indicated by an increase in anti-oxidant activity, suggesting that the *C. sativus* extract may have a neuroprotective role in mitigating toxicity through the suppression of oxidative stress and an increase in the expression of anti-oxidant enzymes (108). In a similar study, the administration of *C. sativus* extract (60 mg/kg, IP, for 6 days) was found to suppress oxidative stress and enhance the recovery of enzyme activity, specifically monoamine oxidase and acetylcholinesterase, in the brain and cerebellum of mice with aluminum-induced impairment of learning and memory (109). Furthermore, there is evidence to suggest that a 3-week treatment involving *C. sativus* extract at a dosage of 30 mg/kg, IP, along with crocin at a dosage of 15–30 mg/kg, IP, exhibited a protective effect against oxidative stress as well as spatial learning deficit and memory damage induced by chronic stress in mice (110). 

Treatment with two doses of *C. sativus* extract (5 and 10 μg/rat for 1 week) after the induction of multiple sclerosis (MS) using intra-hippocampal administration of ethidium bromide (EB) resulted not only in the amelioration of memory deterioration and enhancement of spatial learning, but also in a significant elevation in the levels of agents possessing anti-oxidant properties, products of lipid peroxidation, and activity of enzymes with anti-oxidant properties in the hippocampus of the groups that received treatment. Furthermore, following 7 consecutive days of treatment, the anti-oxidant status was reinstated to a state of normalcy (111). Thioflavine T-based fluorescence of Aβ1-40 measurement indicated that aqueous and methanolic extract of *C. sativus* reduced the aggregation and formation of Aβ fibril (a pathological sign of the onset of Alzheimer’s disease) and thus inhibited memory impairment caused by the destruction of cholinergic neurons in the human brain in a time and concentration dependent manner (112). Furthermore, the extract of *C. sativus* exhibited an augmentation in the transcription of the gene encoding brain-derived neurotrophic factor (BDNF) and the subsequent synthesis of BDNF and cAMP response element binding protein (CREB)(113). *C. sativus *is capable of averting motor dysfunction in 1-methyl-4-phenyl-1,2,3,6-tetrahydropyridine (MPTP)-induced elimination of dopaminergic neurons by upholding the BDNF factor within the neurons (114).

*Crocin*

In cultured rat brain microglial cells, crocin (20 µM) repressed microglial activation induced by LPS-induced inflammation, inhibited nitrite production, inactivated NF-κB signaling, decreased pro-inflammatory cytokines such as TNF-α and IL-1β, and induced apoptosis in the rat hippocampal tissue. These observations suggested that crocin can play a protective role against oxidative stress produced by active microglia cells in the brain (115). Crocin (20 and 10 mg/Kg) exhibited therapeutic effects on ischemia/reperfusion (I/R) induced injury in mice by suppressing oxidant factors and modulating the ultrastructure of cortical microvascular endothelial (CMEC) cells. These findings suggest that the mechanisms underlying the therapeutic actions of crocin involve the inhibition of translocation of G-protein coupled receptor kinase 2 (GRK2) from the cytosol to the membrane, suppression of phosphorylation of extracellular signal-regulated kinase 1/2 (ERK1/2), as well as reduction in the number of cortical microvessels and expression of MMP-9 (69).

In ethanol-induced memory retrieval deficit mice, pre-administration of crocin (50 to 200mg/kg, PO) showed a preventive effect against ethanol-induced learning and memory deficit (116). Treatment with crocin (200 µm) on day 7 day after EAE induction, resulted in suppressed XBP-1/s gen in the spinal cord (117). Astrocyte and oligodendrocyte inflammation and cell toxicity are the main causes of EAE, and syncytin-1 and NO produced in this process were reduced by crocin (118). 

Pretreatment of traumatic brain injury (TBI) animal model with α-crocin (20 mg/kg) reduced proinflammatory cytokines, microglial activation, and brain edema, and improved neurological severity score in mice (119). Crocin administration (150 mg/kg) resulted in a reduction in the release of calcitonin gene-related peptide (CGRP), as well as an improvement in locomotor function and mechanical behavior in rats with spinal cord contusion injury (120). Moreover, administration of *C. sativus *(125 and 250 mg/kg) and crocin (50-200 mg/kg, PO) demonstrated protective effects against ethanol-induced performance deficits, such as suppression of LTP, memory disorders, and learning impairments, both in *in vitro* and *in vivo* conditions, in a dose-dependent manner (121). 

Single dose of *C. sativus *ethanolic extract (125, 250, and 500 mg/kg, PO) enhanced memory acquisition and retrieval, and improved hippocampal synaptic plasticity in ethanol-induced impairments of learning and memory in animal models. Examination of the rat hippocampal dentate gyrus showed that this effect of crocin occurs via antagonized NMDA receptors changing synaptic potentials (122). Treatment with *C. sativus *aqueous extract (0.0025–0.56 g/kg), crocin (50 and 200 ml/kg), and safranal (0.2 ml/kg) for 5 days in rats, was able to prevent scopolamine-induced learning impairment (123). *C. sativus* extract (30 and 60 g/kg) and crocin (15-30 mg/kg) also showed improved retrieval spatial memory and working memory in the novel object recognition test (NORT) and the radial water maze task in rats (98).

Treatment with crocin (15 and 30 mg/kg, IP, for 22 days) on sporadic Alzheimer’s disease induced by intracerebroventricular (icv) STZ in male rats has shown improvement in learning and memory performance. A high dose of crocin (30 mg/kg) antagonized the cognitive deficits and diminished the symptoms of the neurodegenerative disease (124). In ethanol-induced memory impairment, crocin blocked the inhibition of NMDA response by ethanol (125). Crocin showed antihyperglycemic, antihypoinsulinemic, and neuroprotective effects in STZ-induced diabetic rats (126).

It has been suggested that crocin analogs, including crocetin gentiobiose glucose ester and crocetin di-glucose ester, can reduce the effects of alcohol on LTP blocking (127). Oral administration of crocin (100 mg/kg) in STZ (3 mg/kg, icv)-induced diabetic rats, improved spatial memory deficit and decreased oxidative stress (128). A similar study showed that crocin (15, 30, and 60 mg/kg, IP for 6 weeks) administration to rats with diabetes-induced spatial memory impairment via modulating cerebral oxidative damage modified spatial memory in the Morris Water Maze paradigm (129).

Treatment with crocin (15 and 30 mg/kg), in the form of a single injection, in ketamine-induced rats enhanced recognition memory through antagonized NMDA glutamate receptors indicating its anti-oxidant properties (130). Using the object recognition task and a novel version of the radial water maze, prescribing crocin (30 and 15 mg/kg) to scopolamine (0.2 mg/kg)-induced performance deficits animal model, modulated storage and/or retrieval of information. Furthermore, the administration of crocins (15 and 30 mg/kg) effectively mitigated the negative effects of delay-dependent recognition memory deficits in normal rats (98). 

In rats with ketamine-induced retrograde amnesia, crocin (2 mg/kg, IP) exhibited a correlation with the glutamatergic system in the facilitation of passive avoidance memory, effectively ameliorating retrograde amnesia in rats (131). 

Crocin can inhibit bcl-2, Bax, and Caspase-3, which play a major role in the onset of apoptosis in H2O2-induced damaged PC-12 cells (132). In acrylamide (ACR)-induced PC12 cell cytotoxicity, treatment with crocin (10-050 µm) provided cellular protection against apoptosis induced by ACR, partially through the inhibition of intracellular ROS generation (133). In another study, crocin (12.5, 25, and 50 mg/kg, IP) improved histopathological damages in the cerebral cortex and cerebellum regions as well as behavioral symptoms in rats exposed to intra-peritoneal ACR (50 mg/kg)(134).

Amyloid-β and interferon-gamma (IFN-γ) are major stimulators for oxidant factor production such as NO, intracellular ROS, TNF-α and IL-1β, and NF-κB activation in LPS-induced brain microglial cells. Crocin and crocetin reduced microglial cell activity and oxidant factors and reversed the neurodegeneration process in rats (115). Administration of crocin in different doses during 21 days in Wistar rats led to increased production of proteins and brain factors including CREB and BDNF at higher doses≥50 mg/kg, and nerve growth factor (VGF) in 12.5, 25, and 50 mg/kg doses (135).

*Crocetin*

Crocetin gentiobiose glucose ester can protect the hippocampus from the effects of alcohol (127). Pretreatment of dementia animal model with *Nardostachys jatamansi* extract (200 mg/kg), crocetin (25 mg/kg), and selenium (0.05 mg/kg) synergistically improved memory performance through reducing oxidative factors (136). Crocetin also reduced the cytotoxicity effects of beta-Amyloid-(1-42) through modulation of oxidative stress in murine HT-22 hippocampal neuronal cells (137). 

*Safranal*

 In Kainic acid induced-anesthetized rats, the extracellular concentrations of glutamate and aspartate in the rat hippocampus were decreased following pretreatment with safranal (72.75 or 291 mg/kg, IP)(138). IP injection of safranal (727.5, 363.75, and 145.5 mg/kg) in the ischemic hippocampus of mice amended reperfusion in global and focal cerebral ischemia by modulating oxidative stress (139). In an experimental animal model of chronic cerebral hyperfusion, safranal at different doses improved spatial cognition through anti-oxidant activity enhancement (133). Administration of safranal (100 mg/kg, IP) after spinal cord injury reduced the inflammatory cytokines and aquaporin-4 expression which alleviated edema (140). Also, safranal (72.75, 145.5, and 291 mg/kg, IP) showed therapeutic effects against neurodegeneration in rats exposed to quinolinic acid (141). Table 5 presents a summary of the neuroprotective effects exhibited by *C. sativus* and its constituents.

***Effect on metabolic disorders***

*C. sativus*

It was reported that hydro-methanolic extract of *C. sativus *(50 mg/kg, IP) significantly reduced serum glucose and cholesterol levels, and increased insulin levels in healthy male rats (142). Administration of *C. sativus *ethanolic extract to alloxan-induced diabetic rats in comparison with lbutamide, as a standard drug, reduced fasting blood glucose (FBG) levels by regenerating damaged pancreatic islet cells (143). Administration of *C. sativus *(40 and 80 mg/kg, for 4 weeks) decreased TC and LDL but increased HDL in type 2 diabetic rats induced by STZ. Furthermore, body weight and serum TNF-α were increased but serum advanced glycation end products (AGEs) level was decreased. Also, blood glucose levels and glycosylated serum proteins were significantly reduced (144).

Peroxisome proliferator-activated receptor 𝛼 (PPAR𝛼) could be activated by fibrates such as *C. sativus*. Activating these receptors by *C. sativus* may potentially ameliorate the quantity of PPARα agonists that could potentially contribute to an enhanced lipid profile (145). Administration of aqueous and alcoholic extracts of *C. sativus *to STZ diabetic rats decreased total glyceride and very low-density lipoprotein (VLDL) but increased adiponectin (146). 

Administration of *C. sativus *extract (200 mg/kg body weight) to STZ-induced diabetic rats five times a week, resulted in the prevention of weight loss and fasting blood sugar. The level of TNF-α was also decreased in the the kidney, liver, and lens tissues of diabetic rats (147). The therapeutic properties of *C. sativus* in rats with diabetes induced by alloxan resulted in a decrease in FBG and HbA1c levels, as well as an increase in blood insulin levels (148). Treatment with *C. sativus *ethanolic extract (40 mg/kg) and crocin (80 mg/kg) in rats who received a high-fat diet for 12 weeks improved the lipid profile. Crocin also decreased total glyceride and total cholesterol levels (149). *C. sativus *increased the synthesis of plasma liver proteins such as albumin through changes in the function of hepatocytes (150). 

Administration of *C. sativus *extract (100 mg/kg) and fenugreek supplementation (1.4 g/kg) to STZ-induced diabetic rats, resulted in a reduction in the levels of total lipids, total cholesterol, triglycerides, low-density lipoprotein (LDL), and very low-density lipoprotein (VLDL) both in the serum and liver. However, there was an increase in the levels of high-density lipoprotein (HDL) in the serum, as well as an increase in the total protein, serum albumin, globulin contents, and the A/G ratio in the liver. *C. sativus, *and fenugreek also inhibited the reduction of glycogen and total liver protein and led to the preservation of the structural integrity of the liver (151). In another study, administration of *C. sativus *ethanolic extract (200, 400, and 600 mg/kg) in alloxan-induced diabetic rats significantly decreased blood glucose levels and improved lipid profile (152). Ethanolic extract of *C. sativus *alleviated ER stress and protein ubiquitination, induced cell apoptosis, and modulated protein oxidation in hepatic I/R injury (153).

*C. sativus *aqueous extract (200 mg/kg, IP, for 5 weeks) protected the kidney and liver against damage caused by STZ-induced diabetes in rats, due to its anti-inflammatory potential. Also, FBG level was reduced and weight loss was prevented in treated diabetic rats (147). *C. sativus* ethanolic extract administered to STZ-induced diabetic rats, decreased the levels of aminotransferases, ALT and AST as indicators of the hepatocyte intracellular enzymes, ALP and bilirubin as indicators of liver damage, and albumin as liver protein synthetic function. The plant also improved lipid peroxidation in liver tissue including MDA, GSH, GSH-Px, SOD, and CAT. Therefore, due to the anti-oxidant effects, ethanolic extract of *C. sativus *showed hepatoprotective effects in STZ-induced diabetic rats with liver injury (154).

Treatment of obese Wistar rats with *C. sativus *methanolic extract (25, 50, 100, and 200 mg/kg) and crocin (5, 15, 30, and 50 mg/kg) for two months, reduced appetite, body weight, and leptin levels. *C. sativus *and crocin also reduced the volume of adipose tissue and increased insulin sensitivity by diminishing leptin levels (155).

*Crocin*

Crocin (50 or 100 mg/kg, IP, for 150 days) administration in neonatal male Wistar rats with STZ-induced type 2 diabetes, aged 2-5 days, resulted in a decrease in various biochemical factors. These factors include serum glucose, advanced glycation end products, hba1c, triglyceride, total cholesterol, and LDL. Additionally, the level of HDL was increased, and microalbuminuria was reduced in the diabetic model. These changes were utilized in the assessment of the homeostatic model for insulin resistance degrees (156). In the rat model of DZN-induced hepatotoxicity, crocin (12.5 and 25mg/kg/day, IP) inhibited hyperlipidemia through the declined inhibition of ERK performance, and increased LDL receptor expression (157). In a similar animal model, administration of crocin (12.5, 25, and 50 mg/kg/day, IP) reduced caspases, Bax/Bcl-2 ratio, lipid peroxidation, and pathological changes in rat liver, and led to inhibition of hepatotoxicity (158). 

The reduction of blood glucose and lipid peroxidation levels, along with the increase in thiobarbituric acid reactive substance (TBARS) and total thiol (SH) group levels, as well as the decrease in anti-oxidant activity in the kidneys and liver, demonstrated the effects of crocin on hyperglycemia and oxidative stress in a rat model of diabetes induced by STZ. These observations provide evidence for the anti-hyperglycemic and anti-oxidant properties of *C. sativus* in the diabetic state, which can be attributed to crocin (159).

Crocin ameliorated the toxicity induced by cyclophosphamide through the modulation of the anti-oxidant status and inflammatory cytokines (160). The impact of crocin and crocetin on the levels of GPX in the liver, SOD in the liver and kidneys, and to a lesser extent on total anti-oxidant capacity (TAOC) in the heart were documented (161). 

*Crocetin*

High-fructose diet (HFD) feeding and crocetin treatment in male Wistar rats reduced free fatty acid, rectified dysregulation of mRNA expression of adiponectin, TNF-α, and leptin which was probably related to alleviated insulin resistance. These observations may suggest the protective effect of crocetin against insulin resistance (162). Administration of crocetin (2 mg/kg, PO) preserved cellular ATP, inhibited mRNA expression and production of TNF-α and IL-1β in hepatocytes, protected against cellular damage, and increased overall survival cell life in hemorrhagic shock (163).

In rats with pancreatic disorder and dexamethasone-induced insulin resistance, crocetin reduced free fatty acids, triglyceride, and TNF-α, and increased insulin secretion by reinforcing pancreatic islet beta cells (164). Crocetin inhibited insulin resistance and raised hepatic lipoprotein lipase activity in HFD-induced insulin resistance in rats (165).

*Safranal*

Safranal (0.5 mg/kg/day, IP for one month) administration in 2, 10-, and 20-month-old rats increased the activity of anti-oxidant enzymes and reduced the rate of normal aging by suppressing oxidative stress (166). Safranal decreased serum and pancreas TNF-α and IL-1β, and oxidative stress in HFD and STZ-induced DT2 in rats (167). Administration of safranal (20 mg/kg, PO, for 2 weeks) led to dephosphorylation of the insulin receptor via inhibition of protein tyrosine phosphatase 1B (PTP1B), which plays a role in insulin signaling and improved impaired glucose tolerance (168). In *Escherichia* colitis, safranal blocked wild-type F1FoATP synthase exclusively. Notably, αR283D mutant ATP synthase experienced a significant reduction of approximately 50% in its functionality (169). Table 6 presents a summary of the effects of *C. sativus* and its constituents on metabolic syndrome.

***Gastrointestinal diseases***

*C. sativus and Crocin*

Oral administration of 1% *C. sativus *aqueous extract in *Drosophila melanogaster* intestinal immunity, reduced epithelial cell death and ROS production. The anti-inflammatory and anti-oxidant effects of *C. sativus* against intestinal damage led to improved intestinal morphology and increased lifespan of adult flies (170). Administration of aqueous-ethanolic extract of *C. sativus* (60 mg/ml, IP) reduced ileum contractions in guinea pigs stimulated by electrical field stimulation (EFS). The extract was also able to reduce the ileum contractions induced by epinephrine (1 μM), but it did not affect the contraction induction by KCl (300 mm). These observations showed a postsynaptic inhibition-mediated decrease in intestinal contraction (171). Pre-treatment with oral administration of *C. sativus* extract, crocin, and safranal, increased glutathione activity and protected gastric tissue against indomethacin-induced tissue changes in diabetic and non-diabetic rats (172). Treatment of mice exposed to indomethacin with both crocin and pantoprazole also reduced gastric index (mm2) (173).

*Crocetin*

Crocetin administration to 1-methyl-3-nitro-1-nitrosoguanidine (MNNG)-induced gastric adenocarcinoma (AGS) rats inhibited proliferation, induced apoptosis, suppressed Bcl-2, and increased regulation of Bax gene expression as well as increasing lactate dehydrogenase and *anti-oxidant* agent activity. Together, these effects inhibited tumor growth in a dose- and time-dependent manner. In addition, the observations of the MTT assay showed that treatment of normal human fibroblast (HFSF-PI3) cells with crocetin did not lead to these changes. These results suggest therapeutic applications of crocetin in inhibiting gastric adenocarcinoma in humans (174). 

In burn-induced intestinal injury in rats, crocetin (100 and 200 mg/kg) suppressed inflammatory signaling pathways such as NF-κB and polymorphonuclear neutrophil (PMN) accumulation and reduced TNF-α and IL-6 levels. Inhibition of these inflammatory responses led to ameliorating focal necrosis and mucosal ulceration in the damaged small intestine (175). Colonic architecture disorder and diarrhea were the most important disorders that improved after administering crocetin (50 mg/kg, intragastric) to mice. In addition, it reduced the severity of inflammation, lipid peroxidation, NO production, and Th1 and Th2-related cytokines. Therefore crocetin may improve the symptoms of 2,4,6- trinitrobenzene sulfonic acid (TNBS)-induced colitis including epithelium necrosis, inflammatory cell reduction, and distortion of crypts (49).

*Safranal*

The reported findings indicate the presence of anti-oxidant, anti-inflammatory, and anti-apoptotic effects of safranal in countering the occurrence of gastric ulcers induced by indomethacin (176). Safranal exerted a suppressive effect on inflammation and apoptosis in indomethacin-induced gastric ulcers, which can be attributed to its ability to inhibit caspase-3. This particular caspase is categorized as one of the cysteine proteinases that participate in inflammatory processes and apoptosis. Anti-secretory and anti-oxidant effects of safranal against gastric ulcers were also reported (177). The activity of *Helicobacter pylori* was inhibited by the administration of semi-synthetic derivatives of safranal, namely thiosemicarbazonic derivatives and (thiazol-2-yl) hydrazonic, as well as safranal and crocin. These natural components probably inhibit the enzymatic activity of biological processes in *H. Pylori* strains. These compounds also showed anti-parasitic activity of the plant such as bits effect on *Plasmodium *and *Leishmania* (178). The experimental application of N-095, a compound comprising red ginseng, polygala root, saffron, antelope horn, and aloe wood, in rats exhibited a significant safeguarding influence against histamine-induced gastric ulceration (179). Table 7 presents a summary of the gastrointestinal effects exhibited by *C. sativus* and its constituents.

***Respiratory diseases***

*C. sativus*

Administration of *C. sativus* extract with dexamethasone in guinea pigs model of ovalbumin (OVA)-induced asthma increased IFN-γ level, had a stimulatory effect on T-helper 1 cells, and decreased IL-4 production or had an inhibitory effect on T-helper 2 cells leading to improved Th1/Th2 balance (180). The decrease in the count of white blood cells (WBC) and the proportion of neutrophils and eosinophils were observed as a consequence of administration of hydroethanolic extract derived from *C. sativus *(50, 100, and 200 mg/kg) in sensitized animals in a similar study (181).

Administration of hydroethanolic extract from *C. sativus* in animals sensitized with OVA resulted in a decrease in lung pathological changes, including infiltration of eosinophils and lymphocytes in the interstitial space, infiltration of cells in the interstitium, atelectasis, lung congestion, bleeding, and epithelial damage. Additionally, administration of hydroethanolic extract reduced the total WBC count, as well as the number of eosinophils and lymphocytes, which exhibited a comparable or even more potent effect than that of dexamethasone (182). In a similar study, administration of hydroethanolic extract derived from *C. sativus* (0.1, 0.2, and 0.4 mg/ ml) to guinea pigs sensitized with OVA resulted in a decrease in serum levels of endotheline-1 (ET-1) and total protein (TP)(183).

Pretreatment with hydroalcoholic extract of *C. sativus *(50, 100, and 200 mg/kg, IP) in asthmatic rats exhibited a reduction in both the total and differential counts of WBC, red blood cells (RBC), and platelets, indicating that the plant can be used in the treatment of asthma (181). Administration of ethanolic extract of *C. sativus* stigma (100–800 mg/kg, IP), safranal (0.25-0.75 ml/kg, IP.), and crocin reduced the number of citric acid 20%-induced cough in guinea pigs (184). The relaxant effect of *C. sativus *(0.1 and 0.2 g%) and safranal (1.25 and 2.5 mg) on tracheal smooth muscles (TSM) by stimulation of β2-adrenoreceptors and inhibition of histamine (H_1_) receptors was also demonstrated (185). Hydro-ethanolic extract of *C. sativus* (0.15, 0.3, 0.45, and 0.60 g%) showed a relaxant effect on guinea pigs TSM in a dose-dependent manner similar to the effect of theophylline (186). It was shown that the relaxant effect of the extract of saffron on the tracheal smooth muscle of guinea pigs is mediated through a stimulatory effect on β2-adrenoreceptorsor inhibitory effect on histamine (H_1_) or muscarinic receptors (187, 188).

*Crocin*

Administration of crocin (30, 60, and 120 μM/ml) on pre-contracted rat TSM showed a significant relaxant effect through the opening of potassium channels muscarinic receptor blocking and ß2-adrenoreceptors stimulation (189).

Treatment of OVA-sensitized mice with crocin (100 mg/kg), significantly reduced the BALF levels of IL-4, IL-5, and IL-13, inhibited the expression of lung eotaxin, p-JNK and p-ERK genes, lung eosinophil peroxidase, and decreased airway hyperreactivity indicating its anti-asthmatic property (190). Administration of crocin (25 mg/kg/day, PO) in mice with asthma induced by OVA resulted in a decrease in levels of TNF-α, IL-4, IL-13, LDH, and MDA, while levels of SOD and GSH in lung tissue were increased (191).

Administration of crocin (50 mg/kg/day, 3 times/week, for 8 weeks) to cigarette-induced COPD rats inhibited the gene expression and protein production of a nuclear factor erythroid 2-related factor 2 (Nrf2), protein kinase C (PKC), mitogen-activated protein kinase (MAPK), phosphatidylinositol 3-kinase (PI3K) and glutamate-cysteine ligase catalytic (gclc), and prevented lung damage by strengthening the anti-oxidant system (192). Also, oral administration of crocin (50 mg/kg) improved acute lung damage caused by intratracheal injection of LPS by suppressing myeloperoxidase (MPO) activity, preventing lung edema, reducing NO level and iNOS expression, and inhibiting the production of TNF-α and IL-1β (193). Treatment of LPS-induced lung injury in mice with crocin (50 and 100 mg/kg) resulted in the suppression of phospho-iκb expression and the activity of the NF-κB pathway. Furthermore, crocin treatment led to a decrease in the expressions of TNF-α and IL-6 at the protein and mRNA levels, as well as a reduction in the levels of macrophage chemoattractant protein-1 (MCP-1) in the lung tissue (194).

*Crocetin*

Intranasal administration of crocetin (100 μM/day, for 9-10 weeks) in OVA-induced asthma in mice, reduced the number of Treg cells and the levels of Foxp3 and TIPE2, indicating treatment properties of crocetin in asthma (195). 

Administration of crocetin (50 and 100 mg/kg, intragastric injection) before induction of LPS-induced inflammation in mice reduced neutrophil inﬁltration, inhibited thickness of the alveolar wall, intra-alveolar exudation, interstitial edemas, MPO activity and mRNA levels of TNF-α, MCP-1, and IL-6, increased SOD, and improved lung tissue damage (194). 

Administration of crocetin (100 mg/kg, IV) and a trans isomer of sodium crocetinate (TSC) in rats with hypoxic exercise, increased the ability of the lung for O2 diffusion and transport in animal models (196).

Crocetin treatment (50 mg/kg/day, IP, for 2 weeks) in bleomycin-induced sclerotic mice, decreased procollagen (COL1A1) miRNA and ET-1 mRNA levels, and reduced the process of lung tissue fibrosis (197).

*Safranal*

Safranal, administered at varying doses of 0.15, 0.30, 0.45, and 0.60 ml of a solution containing 0.2 mg/ml, exhibited a dose-dependent relaxant effect on the tracheal smooth muscle of guinea pigs, resembling the effect observed with theophylline (186). Administration of safranal (1.25, and 2.5 μg/ml) on guinea pig TSM showed similar effects to chlorpheniramine which indicated an inhibitory effect on histamine H_1_ receptors as a competitive antagonist (198). Studies have shown that the possible mechanisms of safranal (1.25 and 2.5 mg) induced relaxation of TSM include stimulation of β2-adrenoreceptors and inhibition of histamine (H_1_) receptors (198, 199). Furthermore, the cumulative log concentration-response curves of methacholine acquired while the aqueous-ethanolic extract of *C. sativus* (25, 50, and 100 µg/ml) and safranal (0.63-2.5 µg/ml) were present at various concentrations exhibited a noticeable deviation to the right when compared to the methacholine curves generated in the presence of saline. Consequently, these findings clearly suggest the existence of a competitive antagonistic influence exerted by safranal on muscarinic receptors (199).

Safranal decreased TSM responsiveness to methacholine in sensitized guinea pigs through increased IFN-γ and decreased IL-4 and NO levels (200).

Administration of safranal (0.25, 0.50, and 0.75 mg/kg/day, for 28 days) to diabetic rats increased CAT, SOD, and GSH levels in lung tissue and bronchoalveolar lavage fluid (BALF) and therefore protected lung tissue against lung damage caused by diabetes (201). Treatment with safranal (0.1, 0.5, and 1 mM), inhibited IFN-γ and IL-10 secretion and cell viability in the peripheral blood mononuclear cells (PBMC) and increased IFN-γ secretion and IFN-γ/IL-4 ratio indicating its effect on Th1/Th2 balance (202).

Safranal significantly improved the pathological and immunological changes of the lungs and alleviated lung pathological changes. It also reduced serum histamine levels and improved total and differential WBC counts in lung lavage in OVA-sensitized guinea pigs (182).

*Kaempferol*

Oral administration of kaempferol to OVA-sensitized mice, inhibited mucus secretion in bronchial airways cells and suppressed goblet cell hyperplasia (203). Table 8 presents a summary of the respiratory effects exhibited by *C. sativus* and its constituents.

***Renal diseases***

*C. sativus*

The diuretic property of *C. sativus *was shown by increasing blood flow and improving blood circulation (11). In addition, in the realm of diagnosing and treating conditions affecting the glomerulus such as glomerulonephritis or localization of antigen-antibody complexes, *C. sativus *as a safe remedy showed a diuretic effect by increasing renal blood flow (204). In a descriptive study in cats, an aqueous extract of *C. sativus *showed a diuretic effect and increased glomerular filtration rate (205). Treatment with *C. sativus *petal extracts (40 mg/kg, IP) of gentamicin sulfate-induced nephrotoxicity in male Wistar rats-induced kidney failure, resulted in a significant reduction in serum BUN and creatinine levels, as well as improving kidney histological changes, indicating a protective effect of *C. sativus *against kidney failure caused by gentamicin sulfate (206).

Administration of *C. sativus *(10, 40, and 90 mg/kg) increased artery blood flow velocity in kidney arterials due to the lowest dose of *C. sativus *(10 mg/kg) in male Sprague-Dawley rats. It also directly affected endothelial cells and improved their function. While the use of low doses of the plant is recommended as a treatment for ischemic kidneys, higher doses were harmful due to tissue lesions such as acute tubular necrosis or injury (ATN) and glomerulopathy (204). Treatment with hydro-ethanolic extract of *C. sativus *petals (20 mg/kg) in acetaminophen (APAP)-induced acute nephrotoxicity in male Wistar rats significantly decreased serum creatinine and uric acid. These results indicated a protective effect of *C. sativus* on acute nephrotoxicity induced by APAP (207). Treatment with *C. sativus *extract (5, 10, or 20 mg/kg, IP) in male rats with renal inflammation induced by I/R was observed to significantly decrease the levels of MDA and TNF-α, infiltrated leukocytes, as well as the serum concentrations of creatinine and urea-nitrogen. Additionally, the expression levels of intercellular adhesion molecule-1 (ICAM-1) mRNA were down-regulated. This study showed that *C. sativus *can protect the kidney against I/R-induced AKI due to its anti-inflammatory and anti-oxidant effects (208).

Administration of hydro-ethanolic extract of *C. sativus* (for 4 weeks, IP) to middle aged and aged rats, significantly reduced pro-inflammatory cytokine, lipid peroxidation, and oxidant factors, and suppressed inflammatory gene expression in aging rats’ kidneys (209). Administration of *C. sativus *aqueous extract and crocin significantly diminished the oxidative stress caused by renal I/R in rats (210).

*Crocin*

In STZ-induced diabetic male rats, treatment with crocin for two months, lowered blood glucose levels, increased insulin secretion, and improved renal function. In addition, treatment with crocin increased creatinine clearance with proteinuria along with a decrease in serum creatinine and nitrogen levels. It also reduced the content of NOS and LDH activity. Oxidative indices such as MDA and toll-like receptors 4 and IL-6 were decreased and protein expression of NF-κB/p65 was inhibited, but serum anti-oxidants such as SOD, GSH, and CAT were significantly increased (211). Table 9 presents a summary of the renal effects exhibited by *C. sativus* and its constituents.

***Urogenital diseases***

*C. sativus*

Administration of *C. sativus *extract along with *Serenoa repens* (Serenoa) and *Pinus massoniana* (Pinus) reduced inflammation in bacterial or non-bacterial prostatitis and improved its symptoms, including sexual dysfunction such as concomitant erectile dysfunction and urinary tract disorder. The production of ROS in immortalized prostate cells (PC3) was inhibited in LPS-induced prostatitis, and the NFκb and PGE2 pathways were suppressed. The results showed that the combination of *C. sativus* with two other compounds has synergistic anti-inflammatory and anti-oxidant effects in prostatic treatment (212).

Treatment of cadmium-induced infertility and impaired spermatogenesis with *C. sativus* in rats decreased cell division and lipid peroxidation, and increased cell proliferation and Johnsen scores in seminiferous tubules, free serum testosterone, and the number and survival time of sperm in the cauda epididymis (213). 

Administration of *C. sativus *stigma aqueous extract (160 and 320 mg/kg, IP) and crocin (100, 200, and 400 mg/kg) into male rats, increased mounting frequency (MF), intromission frequency (IF), and erection frequency (EF), and decreased mount latency (ML), intromission latency (IL) and ejaculation latency (EL). These observations showed enhanced aphrodisiac activities of the *C. sativus *stigma and crocin similar to sildenafil (60 mg/kg)(214).

*Crocin*

Increased ROS production levels negatively affected the genetic integrity, motility, and fertilization capacity of sperm. Treatment of bovine sperm with crocin (0.5, 1, and 2 mM at intervals of 2-4 hours) significantly reduced ROS production and lipid peroxidation and increased motility and viability. In addition, the number of fragmented cells was decreased. These results indicate increasing fertilization capability of crocin in bovine sperm by modulating the concentration of DNA (215). 

In an experimental animal study, simultaneously, on male Sprague Dawley rats that received cyclophosphamide (15 mg/kg, once a week for 8 weeks) for induction of toxicity in testicular tissue, administration of crocin (10 and 20 mg/kg/day, for 56 days) improved glutathione redox cycle protection and sperm quality, increased hormonal mediators, and reduced caspase-3 activity and the process of apoptosis in testicular tissue. These data suggested the protective effect of crocin on testicular tissue and function against the side effects of cyclophosphamide in a dose-dependent manner (216). Table 9 presents a summary of the urogenital effects exhibited by *C. sativus* and its constituents.

## Discussion

There are various hypotheses to explain the anti-tumor properties of *C. sativus* and its main components, the most important of which include the inhibitory effect on DNA and RNA synthesis without an inhibitory effect on proteins, and the inhibitory effect on chain reactions that eventually lead to free radical production, deactivate topoisomerase II with carotenoids, and natural metabolic functions such as conversion carotenoids to retinoids (1). It is explained that *C. sativus* can be used as an anti-cancer agent by the following mechanisms: inhibition of the cell cycle by targeting the DNA sequence and modulating gene expression, which leads to cessation of cell proliferation in the early stages and activation of apoptosis, which leads to the death of cancer cells. *C. sativus* has a protective effect on cancer through induction of apoptosis, inhibition of cell proliferation, and inflammatory and anti-oxidant activities (31). *C. sativus* also plays an important role in preventing tumor progression by regulating some genes, such as p53, prb, Bcl-2 family, and their protein products, which play a key role in cell division and apoptosis. Crocin induces apoptosis, reduces cell invasion, migration, and adhesion by up-regulating e-cadherin expression, and has anti-metastatic potential. Crocetin inhibits proliferation cells by inhibiting glycoprotein and polyamine synthesis, modulates oxidant/anti-oxidant balance, down-regulates the proinflammatory cytokines, and inhibits cyclooxygenase-2 (COX-2) expression in cancer cells (1). Safranal by inhibiting proliferation and apoptosis induction in cancer cells, can be used as a natural treatment for cancer, especially colorectal cancer (217).

*C. Sativus* can be used to treat patients who suffer from hypertension through a reduction in heart rate and contractility via Ca^2+^ channel blockage (54). *C. Sativus* probably reduces the incidence of ventricular arrhythmia by reducing electrical conductivity, prolonging APD, and reducing sensitivity (55). *C. sativus* through stimulation and production of NO leads to the strengthening of the protective function of the AV node against supraventricular arrhythmia (57). *C. sativus* through the anti-oxidant property, showed a protective effect against myocardial I/R injuries (53). Crocin also showed a protective effect on arrhythmias caused by ischemic heart disease (60) and showed prevention of heart disease due to strengthening the anti-oxidant defense system (61). Crocetin possesses the potential to serve as a pharmacological agent in the clinical setting for the management of hypertrophy (74). Furthermore, it exerts an influence on atherosclerosis using its anti-oxidant activity, as well as through its capacity to inhibit the inflammatory response and the p38 MAPK signaling pathway (65). The anti-inflammatory and anti-oxidant characteristics of crocetin may exhibit a safeguarding influence over injuries associated with myocardial I/R (71). Safranal has been demonstrated to diminish MSBP (58) and exhibited a protective effect on myocardial I/R injuries using heightened phosphorylation of Akt/GSK-3β/eNOS, curbed expressions of IKK-β/NF-κB proteins, and potential that counteracts apoptosis (78).

Various studies have proven the antidepressant, anti-anxiety, and anti-seizure effects of *C. sativus* (leaves and stigma) and its major components. Although the mechanistic pathway of the anti-anxiety effect of *C. sativus* and its derivatives has not yet been determined, studies suggest that the flavonoids present in *C. sativus* interact at the benzodiazepine site in the GABA-A receptor which may lead to anti-anxiety effects. Based on *in vitro* and *in vivo* studies, *C. sativus *and its constituents showed anti-inflammatory, anti-oxidant, and neuroprotective activities. These properties are due to the interaction with GABA, cholinergic, glutamatergic, and dopaminergic systems and can be a strategic treatment in neurological disorders such as Alzheimer’s. The proposed mechanisms for anti-Alzheimer’s properties of crocin are inhibition of neurons in the hippocampus via antagonizing NMDA receptor, and the production, accumulation, and formation of amyloid plaques (125). *C. sativus* and its constituents can reduce ethanol, scopolamine, ketamine, morphine, and apomorphine-induced memory acquisition and learning impairment. The plant antagonizes different causes of memory impairment and other neurodegenerative disorders including lipids, proteins, and nucleic acids degradation due to decreased oxidant agents in neurotransmitters/neurotrophin systems (218, 219). *C. sativus* can be also used in the treatment of multiple sclerosis due to its anti-oxidant properties (90). The constituents of *C. sativus*, namely crocin and crocetin, offer neuroprotection through the reduction of various neurotoxic molecules produced by activated microglia (115). Consequently, there has been consideration of negative regulators of microglial activation as potential therapeutic candidates for addressing neurodegenerative conditions, exemplified by Alzheimer’s and Parkinson’s diseases (115).

*C. sativus *showed protective effects on the liver suggesting its potential role in the treatment of liver disorders (220). *C. sativus *can be considered a protective agent in the liver and kidneys against inflammatory processes caused by hyperglycemia (147). *C. sativus *supplements and their active ingredients such as crocin can be used as a therapeutic strategy in the treatment of fatty liver in HFD-induced obese rats. Possible mechanisms of biochemical and histopathological treatment include liver enzyme modulation and restricted fatty infiltration in hepatocytes leading to the liver returning to normal size (221). Crocin may be considered a novel protective agent in hyperlipemia through modulating of ERK pathway and increase of LDL receptor expression (157). The anorectic and anti-obesity properties of *C. sativus *and crocin can be used clinically in the prevention and treatment of obesity (155). *C. sativus *can increase the level of insulin secretion from pancreatic beta cells and can be considered in the treatment of diabetes in the future (142). Due to the anti-oxidant effects, *C. sativus *showed hepatoprotective effects in diabetic rats with liver injury (154). The findings showed that anti-hyperglycemic and anti-oxidant properties of *C. sativus* in diabetic patients are due to crocin (159). The radical scavenging activity of active constituents of *C. sativus*, such as crocin and safranal showed the highest anti-oxidant activity of crocin at concentrations of 500 and 1000 ppm in ethanol solution of 48 to 64%, respectively. However, at the same concentrations, safranal showed lower radical scavenging activity. Therefore, the anti-oxidant properties of *C. sativus* are related to the synergistic effect of crocin and safranal but are mainly due to crocin (222).

Various studies have shown the potential therapeutic effects of *C. sativus* and its constituents such as crocin on a wide range of digestive disorders such as ulcerative colitis, gastrointestinal cancer, and peptic ulcer (5). *C. sativus* affects digestive disorders due to its anti-oxidant and smooth muscle relaxant properties (171, 172). Crocin is converted to crocetin by hydrolysis and then can be converted to mono- and di-glucuronide conjugate metabolites after absorption by the intestine (149). Percentage uptake of crocin in different parts of the gastrointestinal tract was reported as 13.81% in the duodenum, 9.89% in the jejunum, 10.07% in the ileum, and 10.04% in the colon. Also, the degradation of crocin in these parts was 13.01%, 10.11%, 9.95%, and 10.45%, respectively. The aforementioned findings substantiated that crocin does not possess the complete capability to be assimilated across the entirety of the gastrointestinal tract (223). After oral administration, crocin is not present or accumulates in plasma, and its hydrolyzed metabolite is absorbed into the blood as crocetin and is excreted predominantly in the intestine (224). Oral administration of crocetin, its crocetin-monoglucuronide, and crocetin-diglucuronide exist in free and intact forms in plasma. However, no glycoside forms of crocin were found in the blood (225). It was shown that the pharmacokinetic properties of crocin *in vivo* are mainly related to crocetin (226). The therapeutic applications of crocetin were suggested for inhibiting gastric adenocarcinoma in humans (174).

The therapeutic properties of *C. sativus* and its constituents have been known since ancient times and have been further supported by modern pharmacological research. These effects have been observed in the context of lung diseases, specifically in terms of antitussive properties (184), relaxation of the tracheal smooth muscle, stimulation of β2-adrenoceptors, inhibition of histamine (H1) receptors in the tracheal smooth muscle (185, 227), as well as anti-inflammatory and immunomodulatory activities (4). This suggests that *C. sativus* and its constituents may have potential therapeutic applications in the treatment of lung diseases. The effects of *C. sativus *and its compounds on asthma and chronic pulmonary diseases (COPD) were also reported (228). *C. sativus *and its derivatives can be used as antitussive effect agents probably due to airway dilation property (184). The TSM relaxant effects of *C. sativus *and safranal are probably mediated by stimulation of β2-adrenoreceptors and inhibition of histamine (H_1_) and muscarinic receptors (185, 199). The suppressive properties of *C. sativus* on inflammation are likely facilitated through the reduction of inflammatory cytokines as well as the overall and distinct counts of WBC within the lung (182). *C. sativus* has been observed to diminish the levels of IL-4 while concurrently augmenting the secretion of IFN-γ and the IFN-γ/IL-4 ratio, thereby indicating its influence on the balance between Th1 and Th2 (200, 202). Crocin strengthens the anti-oxidant system (192), crocetin inhibits Treg cells, Foxp3, and TIPE2 (195), and kaempferol inhibits the effect on mucus secretion in bronchial airway cells and goblet cell hyperplasia (203) which could affect asthma treatment.

The diuretic property of *C. sativus *was shown by increasing blood flow and improving blood circulation (11). In addition, in the treatment of glomerulus diseases such as glomerulonephritis or localization of antigen-antibody complexes, *C. sativus *as a safe remedy showed a diuretic effect by increasing renal blood flow (204). *C. sativus *indirectly improves the function of the cardiovascular system by strengthening vascular blood flow (204). The findings indicated the protective effect of *C. sativus* against kidney failure caused by gentamicin sulfate (206) and acute nephrotoxicity induced by APAP (207). *C. sativus *possesses the capability to safeguard the kidney from I/R-induced AKI by its anti-inflammatory and anti-oxidant properties (208). In addition, crocin inhibits the process of fibrosis in kidney tissue by inflammatory and fibrotic cascade activation as well as free radical scavenging and anti-oxidant defense system boosting (211).

**Table 1.** Traditional uses of *Crocus sativus* in ancient times

**Systems**	**Effects**	**Ref.**
Cardiovascular System	Cardiac tonic	(30)
	Maintaining heart tone and heart palpitations, improving blood flow, proper nutrition to the heart, antithrombotic and thrombolytic activity	(41)
Respiratory System	Cough relief	(24, 44)
	Sore throat relief	(24)
	Maintaining lung tone, improving respiratory function, and treating asthma	(41)
Gastrointestinal tract	Carminative	(30)
	Clearing the liver of bile	(44)
	Jaundice and bile purification	(26)
	Hepatic obstruction, prevention of gastro-hepatic disorders, treating liver obstruction	(41)
Urinary Tract and Kidney	Diuretic	(30)
Central nervous system	Nervous tonic, stimulant, sedative, relaxant, anti-stress, and anti-anxiety	(30)
	Euphoria	(40)
Reproductive system	Emmenagogue and lactogogue	(30)
Immune system and infections	Febrifuge, otitis, and wounds	(24)
	Diaphragmitis	(44)
Skin	Revitalize facial skin	(44)
Eye	Eye inflammation	(44)
General Effects	Diaphoretic	(30)

**Table 2:** Major components of *Crocus sativus*

**Major compounds**	**IUPAC name**	**Chemical**	**Formula**	**MW**	**Ref.**
Crocetin	(2E,4E,6E,8E,10E,12E,14E)-2,6,11,15-tetramethylhexadeca-2,4,6,8,10,12,14-heptaenedioic acid	Metabolite of crocin	C_20_H_24_O_4_	328.4	(21)
Crocin	bis[(2S,3R,4S,5S,6R)-3,4,5-trihydroxy-6-[[(2R,3R,4S,5S,6R)-3,4,5-trihydroxy-6-(hydroxymethyl)oxan-2-yl]oxymethyl]oxan-2-yl] (2E,4E,6E,8E,10E,12E,14E)-2,6,11,15-tetramethylhexadeca-2,4,6,8,10,12,14-heptaenedioate	Glucosyl esters of crocetin	C_44_H_64_O_24_	976.96	(21)
Kaempferol	3,5,7-trihydroxy-2-(4-hydroxyphenyl)chromen-4-one	Flavonoid compound	C_15_H_10_O_6_	286.23	(22, 23)
Picrocrocin	(4R)-2,6,6-trimethyl-4-[(2R,3R,4S,5S,6R)-3,4,5-trihydroxy-6-(hydroxymethyl)oxan-2-yl]oxycyclohexene-1-carbaldehyde	Terpene-glucoside of safranal	C_16_H_26_O_7_	330.37	(21)
Quercetin	2-(3,4-dihydroxyphenyl)-3,5,7-trihydroxychromen-4-one	Aglycone form of flavonoid glycosides	C_15_H_10_O_7_	302.236	(22, 23)
Safranal	2,6,6-trimethylcyclohexa-1,3-diene-1-carbaldehyde	Monoterpene aldehyde	C_10_H_14_O	150.21	(21)

**Table 3:** Anti-cancer effects of *Crocus sativus* and its constituents in experimental studies

**Compound**	**Dose**	**Study design**	**Possible mechanism and effect**	**Ref.**
Aqueous ext.	25-100 mg/kg, p.o., 5 days	CIS, MMC, and urethane-induced genotoxicity in mice	Inhibited genotoxicity, protective effect against urethane	(35)
	0.2-0.8 mg/ml	HepG-2 and Hep-2 cell lines	Cytotoxic effect on HepG-2 and Hep-2 cell lines	(25)
	0.05-4 mg/ml	Human transitional carcinoma cell, mouse fibroblast	Inhibited growth of TCC 5637 and normal L929 cell lines	(27)
	0.1-0.8 mg/ml	Human breast carcinoma cells	Inhibited MMP gene expression in treated MCF-7 cells	(28)
	200 mg, p.o., 10 weeks	DMBA-induced skin carcinoma in mice	Reduced formation and size of skin papillomas, potentiated cellular defense systems	(32)
	50-500 mg/kg, p.o., 2 weeks	DMBA-induced mice skin papillogenesis/carcinogens	Reduced papilloma, phase II GST, GPx, CAT, and SOD	(33)
	50 mg/kg, IP, 5 days	Cisplatin-induced toxicity in rats	Decreased toxicity by sulfhydryl compounds	(34)
	25-100 mg/kg, p.o., 5 days	CMC and urethane-induced chromosomal damage in mice	Inhibited genotoxicity, exerted protective effect against urethane	(35)
Ethanolic ext.	0.2-2 mg/ml	Human cancer cell lines (HepG2 and HeLa cell lines)	HeLa and HepG2 cells death, decreased malignant cell viability	(24)
	100 mg/kg, p.o., 28 days	Human lung cancer (A549 and H446)	Caspase-8- caspase-9- caspase-3-mediated cell apoptosis	(29)
Aqueous- ethanolic ext.	75-300 mg/kg, p.o., 22 weeks	DEN‐induced liver cancer in rats	Inhibited cell proliferation, apoptosis, oxidative damage	(31)
	100-300 mg/kg, p.o., 10 days	Prostate cancer cell lines (PC3 and 22rv1)	Reduced N-cadherin, ß-catenin expression, PCA cell invasion and migration, increased E-cadherin expression,	(30)
	100 g/ml	MCF-7 and MDA-MB-231 breast cancer cell	Anti-proliferative effect irrespective of T-cell glycosylation	(44)
Crocin	10 μg/L	Human pancreatic cancer cell line	Inhibited growth and apoptotic cell death	(26)
	250 and 500 µg/kg	Melanoma metastasis induction by B16f-10 cells in c57bl/6 mice	Reduced cell invasion, migration, and adhesion by up-regulating E-cadherin expression	(39)
	400 mg/kg, IP, 7 days	Colon adenocarcinoma in rats	Cytotoxic effect on cultured tumor cells, decreased cytoplasmic vacuole-like areas	(40)
	25 mg/ml, i.v., 5 days	Mice bearing C26 colon carcinoma	Anti-tumorigenic activity	(41)
	800 mg/kg, IP, 7 days	NMU-induced breast and gastric cancers in rats	Suppressed tumor growth and p53-dependent in p21Cip1, down-regulated cyclin D1-induced cell cycle arrest	(42)
	100 mg/kg, i.g., 20 days	NMU-induced breast cancer in rats	Decreased tumor volume, latency period and tumor number, EDA and tumor number	(43)
	1.5-6 mg/ml	MCF-7 cells (a type of breast cancer cell line)	Increased apoptosis in MCF-7 cell line	(45)
Crocetin	*In-vivo*: 50-200 µM, 72 hr *In-vitro*: 4 mg/kg, p.o., 30 days	Pancreatic cancer cells MIA-paca-2 and MIA-paca-2 in Xenograft mouse model	inhibited proliferation, altered Cdc-2, Cdc-25C, Cyclin-B1 and EGF receptors Increased apoptosis, inhibited carcinoma cell proliferation	(38)
	1 and 10 µM	Breast cancer cells (MDA‐MB‐231)	Reduced gelatinase activity, MDA‐MB‐231 cell invasiveness via MMP expression down-regulation	(46)
	50 mg/kg, IP, 3 weeks	Benzo(a)pyrene-induced pulmonary adenoma in mice	Inhibited proliferation cells. glycoprotein and polyamine synthesis	(47)
	20 mg/kg, p.o., 4 weeks	Benzo(a) pyrene-induced lung carcinoma in mice	Free radical scavenging, decreased pathological changes	(48)
	50 mg/kg, i.g., 8 days	TNBS-induced colitis in mice	Reduced NO and iNOS levels, down-regulated NF-κB	(49)
	0.1 mg/day, p.o., 10 weeks	AFB1-induced hepatotoxic lesions in rats	Reduced serum AST, ALT, ALP, and GGT, elevated GSH S-transferase activity	(50)
	1- M, p.o., 35 days	MCA-induced uterine cervical cancer in mice	Attenuated MDA, PMN, IL-1, TNF-α, NO3, and mRNA of COX-2 in Hela cells	(51)
Safranal	72.75 and 363.75 mg/kg, IP	MMS-induced DNA damage in mouse organs	Suppressed DNA damage and repressed genotoxic potency of MMS	(52)

AFB1: aflatoxin B1, ALP: alkaline phosphatase, ALT: alanine aminotransferase, AST: aspartate aminotransferase, CAT: catalase, CIS: cisplatin, COX-2: cyclooxygenase-2, DEN: diethyl nitrosamine, DMBA: 7-12 dimethyl benz[a] anthracene, EDA: aminopeptidase, EGF: epidermal growth factor, ext.: extract, GGT: γ-glutamyl trans, GPx: glutathione peroxidase, GST: glutathione-S-transferase, MCA: methylcholanthrene, MMC: mitomycin-C, MMS: methyl Methanesulfonate, NF-κB: nuclear factor-κB, NMU: N-nitroso-N-methylurea, SOD: superoxide dismutase, CMC: Cisplatin, mitomycin-C

**Table 4:** Cardiovascular effects of *Crocus sativus* and its constituents in experimental studies

**Compound**	**Dose**	**Study design**	**Possible mechanism and effect**	**Ref.**
Aqueous ext.	50-00 mg/kg, p.o., 7 days	Ventricular arrhythmias induced by heart I/R, rats	Reduced electrical conductivity, ventricular arrhythmia, prolonging APD	(55)
	2.5-10 mg/kg, i.v., 5 weeks	Normotensive and hypertensive rats	Reduced MSBP	(59)
Aqueous-ethanolic ext.	0.1-5.0 mg%, intracoronary	Isolated guinea‐pig heart	Inhibitory effect on calcium channel	(54)
	2 g/400 ml, p.o., 6 weeks	I/R injuries in isolated rabbit heart	Activated GPX, decreased lipid peroxidation, p38 activity, and infarct size restoration of Akt and 4EBP1 phosphorylation	(53)
	200 mg/kg, p.o., 5 weeks	L-NAME-induced hypertensive rats	Reduced cross-section area, media thickness, and elastic lamellae of aorta, hypertension	(56)
	0.19 mg/L, intracoronary	Rabbit atrioventricular node electrophysiology	Decreased AV node rhythmicity, increased AVCT and FRP facilitation, and the magnitude of fatigue	(57)
Crocin	20 mg/kg, IP, 3 weeks	I/R-induced cardiac arrhythmias in rats	Increased antioxidant systems, protected against cardiac reperfusion arrhythmias	(60)
	10-40 mg/kg, p.o., 21 days	I/R model of isolated rat heart	Antioxidant capacity, prevented cardiac dysfunction and myocardial infarction	(61)
	10-40 mg/kg, p.o., 3 weeks	I/R model of isolated rat heart	Improved cardiac dysfunction, reduced infarct size	(61)
	1 and 10 µM	Bovine aortic endothelial cells	Increased bcl‐2/bax ratio and expression, inhibited aortic endothelial cell apoptosis and atherosclerosis	(62)
	12.5-50 mg/kg, 4 weeks	Diazinon-induced sub-chronic toxicity, rat heart	Reduced SBP, elevated heart rate	(63)
	60 mg/kg, i.v.	Hemorrhagic shock-induced oxidative stress	Attenuated oxidative stress, protected organs from damages	(64)
			Alleviated tissue injuries, increased nuclear translocation of p65 and IkBα phosphorylation	(64)
Crocetin	25-50 mg/kg, p.o., 10 weeks	High cholesterol diet-induced atherosclerosis in rats	Improved lipid profile, aortic lesion, decreased TNF-α, IL-6, MDA, increased SOD, down-regulated p38 MAPK	(65)
	1.0 μM	AGEs-induced VSMCs migration	Decreased AGEs-induced VSMCs migration, RAGE expression, RAGE, TNF-α, IL-6, and MMP-2/9 activities	(66)
	1 μM	Angiotensin II-induced VSM ERK1/2 activation	Blockade of L-type calcium channel	(67)
	0.01, 0.1 and 1 μM	Vascular endothelial cells induced by old age	Reduced AGE-induced BEC, leukocyte adhesion to BEC, ICAM-1 protein, down-regulated MMP	(68)
	1 to 3 μM	VEGF-induced angiogenesis	Reduced VEGF-induced tube formation by HUVECs and migration of HRMECs, p38 and protected VE-cadherin	(70)
	50 mg/kg	Myocardial injury in I/R rat model	Decreased infarct size, CK-MB, MDA, and TNF-α, Increased total SOD and IL-10	(71)
	25, 50 mg/kg, i.g., 15 days	Norepinephrine-induced cardiac hypertrophy in rats	Improved antioxidant enzymatic activities and myocardial pathological changes	(72)
	50 and 100 mg/kg, i.g., 15 days	Overload pressure-induced cardiac hypertrophy in rats	Increased activity of Na^+^, K^+^ -ATPase, Ca^2+^, Mg^2+^ -ATPase, inhibited MMPs activity, reduced cardiac hydroxyproline	(73)
	10-50 mg/kg, p.o., 1 week	AB-induced cardiac hypertrophy, C57/B6 mice	Inhibited MAPK/MEK/ERK1/2 pathway and cardiac hypertrophy	(74)
	0.1-10 μM, p.o., 8 weeks	Hypercholesterolemia in rabbits	Restored thoracic aorta EDR, increased vessel eNOS activity, elevated NO level	(75)
	0.1 and 1 μM	Angiotensin II-induced VSM cell proliferation, bovine	Inhibited phosphorylation and ERK1/2activation, nuclear translocation of activated ERK1/2	(76)
	0.01, 1 and 10 μM	Angiotensin II-induced VSM cell proliferation in bovine	Inhibited angiotensin II-induced cell-cycle progression by arresting the cells in the G0/G1 phase and activation of ERK1/2, decreased ROS, increased SOD activity	(69)
Safranal	1-4 mg/kg, IP, 5 weeks	DOCA-salt-induced hypertensive rats	Reduced MSBP, antihypertensive effects	(58)
	0.1-0.5 ml/kg	I/R heart injury in rats	Enhanced Akt/GSK-3β/eNOS phosphorylation, suppressed IKK-β/ NF-κB expressions, apoptosis	(78)

AB: aortic banding, AGEs: advanced glycosylation end products, APD: action potential duration, BEC: bovine endothelial cells, CK-MB: creatine kinase-MB, DOCA: desoxycorticosterone acetate, eNOS: endothelial nitric oxide synthase, ext.: extract, I/R: ischemia-reperfusion, ICAM-1: intercellular adhesion molecule-1, L-NAME: L- NG-nitro-arginine methyl ester, MAPK: mitogen‐activated protein kinase, MDA: malondialdehyde, MEK/ERK1/2: extracellular signal‐regulated kinase‐1/2 pathway, MMP: matrix metalloproteinase, MSBP: mean systolic blood pressure, NO: nitric oxide, ROS: reactive oxygen species, SBP: systolic blood pressure, SOD: superoxide dismutase, TNF-α: tumor necrosis factor-alpha, VSMCs: Vascular smooth muscle cells

**Table 5:** Neuroprotective effects of *Crocus sativus* and its constituents in experimental studies

**Compound**	**Dose**	**Study design**	**Possible mechanism and effect**	**Ref.**
Aqueous ext.	15 mg/kg	Forced swimming test in mice	Antidepressant effect, decreased immobility time	(80)
	50-450 mg/kg, IP, 5 days	Morphine-induced mice memory impairment	Increased time latency, attenuated morphine-induced memory impairment	(101)
	10-50 mg/kg, IP, 5 days	Morphine-induced inhibition of SLM in rats	Inhibited morphine-induced impairments of SLM	(102)
	100 mg/kg, p.o., 7 days	MCAO model of acute cerebral ischemia in rats	Decreased neuronal cell death and antioxidant effect	(91)
	60 mg/kg, IP, 3 weeks	STZ-induced rat cognitive impairment	Prevented cognitive deficits, attenuated learning and memory impairment	(93)
	0.08, 0.32, 0.56 and 0.80 g/kg, IP	PTZ-induced seizure in mice	Increased the latency of convulsions, decreased duration of tonic seizures	(84)
	200 mg/kg, p.o., 45 days	Aluminum chloride-induced neurotoxicity in mice	Up-regulated BCL-W, INPP4, and R-spondin, alleviated oxidative stress	(108)
	40, 80, 160 mg/kg, IP, 21 days	Forced swimming test in rats	Antidepressant effect, reduced immobility time, increased BDNF, CREB, and p-CREB in the hippocampus	(113)
	2.5-560 mg/kg, IP, 5 days	Scopolamine-induced learning impairment	Reduced latency time, inhibited acquisition/performance activity,	(123)
Ethanolic ext.	30 mg/day, p.o., 6 weeks	Mild to moderate depression, clinical trials	Possible therapeutic effect on mild to moderate depression	(82)
			Antidepressant effect	(83)
	0.2, 0.8, 1.4 and 2.0 g/kg, IP	PTZ-induced seizures in mice	Delayed onset of tonic convulsions, decreased duration of tonic seizures	(84)
	250 and 500 mg/kg	PTZ-induced seizures in rats	Delayed onset and duration of convulsions, reduced seizure duration	(85)
	500 mg/kg, p.o., 21 days	EAE in C57bl/6 mice	Prevented symptomatic EAE, inhibited leukocyte and oxidative stress markers in CNS	(90)
	250 mg/kg, p.o.	Ethanol-hippocampal LTP in rats	Prevented LTP-suppressing action of ethanol in the hippocampus	(95)
	250 mg/kg, p.o., 60 min	Acetaldehyde-induced LTP inhibition in rats	Prevented inhibition of LTP, aversive effects of ethanol and acetaldehyde	(103)
	1-250 μg/ml, IP, 7 days	Healthy adult and aged male mice	Reduced lipid peroxidation, caspase-3 activity, improved learning and memory,	(106)
	1 mg/kg, IP, 5 days	Exposed rat brain to 3-NPA	Antioxidant and neuroprotective against neurodegenerative disorders	(105)
	5 and 10 μg, i.c., 7 days	Ethidium bromide-induced rat memory impairments	Improved learning and memory and hippocampus oxidative stress	(111)
	125-0500 mg/kg, p.o.	Ethanol-induced learning memory impairments	Improved memory, HSP, synaptic potentials, antagonized NMDA receptors	(122)
Aqueous- ethanolic ext.	0.2-0.8 ml/kg, IP	Forced swimming test in mice	Decreased immobility time, dopamine and norepinephrine uptake, increased activity of stereotypic, swimming climbing time	(79)
	15-100 μg/ml, p.o., 30 days	Antioxidant activity measurement *in vitro*	Inhibited Aβ fibrillogenesis, higher values of TEAC and FRAP	(92)
	0.22 and 2.2 μg/ml, 1 month	Amyloid β-induced toxicity in 5XFAD Mice	Increased SP, and reduced neuro-inflammation associated with Aβ pathology in the brain	(94)
	50-200 mg/kg IP, 7 days	Allodynia and hyperalgesia-induced CCINP rats	Alleviated behavioral manifestations of neuropathic pain	(96)
	200 mg/kg, IP, 7 days	CCI model on the left sciatic nerve in rats	Improved MDA and GSH, cytokines, and apoptotic pathways	(97)
	30 mg/kg, IP, 3 days	D-galactose and NaNO2-induced mice memory defect	Prevented and improved amnesia	(99)
	10-200 μg/ml	*In vivo* intracellular recording study	Decreased evoked PSPs, isolated NMDA and non-NMDA of PSPs, kainate-induced depolarization	(100)
	30 and 60 mg/kg, p.o., 24 hr	Scopolamine-induced rat performance deficits	Improved performance deficits in the step-through passive avoidance test	(104)
	0.125−64 μM	*In vitro* enzymatic and molecular docking study	Moderate AChE inhibitory activity	(107)
	60 mg/kg, IP, 6 days	Aluminum-exposed adult mice	Improved memory impairment, AChE and BuChE, activated brain MAO isoforms	(109)
	30 mg/kg, IP, 21 days	Chronic stress-induced SLM deficits in rats	Increased antioxidants, decreased total antioxidant reactivity capacity	(110)
Crocin	15 and 30 mg/kg, IP, 21 days	Chronic stress-induced SLM deficits in rats	Increased antioxidants, decreased total antioxidant reactivity capacity	(110)
	50-600 ml/kg, IP	Forced swimming test in mice	Decreased immobility time, dopamine and norepinephrine uptake, increased stereotypic activity, swimming climbing time	(79)
	5-20 mg/kg, p.o., 30 days	Cognitive impairments in mice	Increased seizure threshold, improved PTZ‐induced kindling, cognitive functions	(86)
	10 μM	Ethanol-induced memory impairment	Blocked inhibition of NMDA response by ethanol	(125)
	15 and 30 mg/kg, IP, 3 weeks	Learning and memory impairments in STZ-induced diabetic rats	Improved learning and memory, antihyperglycemic, antihypoinsulinemic, and neuroprotective effects	(126)
	30 mg/kg, IP, 15 days	MPTP-induced Parkinson's disease mice model	Improved Parkinson’s disease complications, substantia nigra cell death	(114)
	50-200 mg/kg, p.o.	Ethanol-induced learning impairment in mice	Prevented impairment of learning and memory	(116)
	200 µM, 7 days	EAE mice, spinal cords	Improved ER stress, inflammatory gene expression, myelination, axonal density, T cell and macrophage activation, neurobehavioral deficits	(117)
	20 mg/kg	Traumatic brain injury in mice	Improved NSS and brain edema, decreased microglial activation and release of several pro-inflammatory cytokines, decreased cell apoptosis, increased Notch activation	(119)
	150 mg/kg, IP, 2 weeks	SCC-induced chronic pain in rats	Decreased CGRP	(120)
	10-30 μM, p.o	LTP induction in CA1 hippocampal area in rats	Prevented LTP-suppressing action of ethanol in the hippocampus	(121)
	15 and 30 mg/kg, IP, 5 days	Scopolamine-induced spatial memory in rats	Improved recognition memory deficits, information retrieval, and scopolamine-induced performance deficits	(98)
	15 and 30 mg/kg, IP, 22 days	STZ-induced sporadic Alzheimer's in rats	Antagonized cognitive deficits, attenuated learning and memory impairment	(124)
	51.2 nM	Ethanol-induced hippocampal impairment	Prevented impairment of hippocampal synaptic plasticity	(127)
	100 mg/kg, p.o., 21 days	STZ-induced SMD and oxidative stress in rats	Improved cognitive performance, reduced MDA, elevated total thiol and GPx activity	(128)
	15-60 mg/kg, IP, 6 weeks	STZ-induced SMD and oxidative damage in rats	Improved memory dysfunction, antidiabetic and antioxidant activity	(129)
	100 mg/kg, IP, 3 days	Ketamine–induced rat behavioral deficits	Attenuated schizophrenia-like behavioral deficits by non-competitive NMDA receptor	(130)
	2.5 and 10 mg/kg, IP	Ketamine-induced rat retrograde amnesia	Improved passive avoidance memory amnesia interaction with glutamatergic system	(131)
	10, 20, and 50 μM	ACR-induced PC12 cell cytotoxicity	Inhibited Bcl-2 down-regulation, regulated Bax, decreased apoptosis, inhibited ROS	(133)
	12.5-50 mg/kg, IP, 21 days	Acrylamide-induced neurotoxicity in rats	Improved behavioral index, histopathological damages, decreased MDA, elevated GSH	(134)
	12.5-50 mg/kg, IP, 21 days	Healthy male rat hippocampus	Antidepressant-like effect, increased VGF, p-CREB, and BDNF	(135)
	100 mg/kg	PC-12 cells	Prevented dentate gyrus LTP inhibition, N-SMase activation, decreased ceramide release	(132)
	51.2 nM, cerebrventricular	Ethanol-induced hippocampal impairment	Prevented hippocampal synaptic plasticity impairment	(127)
	10–50 μM	Acrylamide-induced cytotoxicity in PC12 cells	Inhibited ROS, protected cells apoptosis	(133)
	50 and 200 ml/kg, IP, 5 days	Scopolamine-induced learning impairment	Inhibition of impaired acquisition/performance activity, reduced latency time	(123)
	5, 10, 20 M	LPS-induced inflammation, cultured brain microglial cells	Reduced NO, TNF-α, and IL-1β production, inactivated NF-κB signaling, induced apoptosis	(115)
Crocetin	0.1, 1, 10 M	Angiotensin II-induced VSMC proliferation	Suppressed G0/G1 phase arresting cells, ERK1/2 activation and ROS, increased SOD	(69)
	1 - 50 μM	*In vivo* intracellular recording study	Improved evoked PSPs and glutamate and NMDA-induced membrane depolarization	(100)
	25 μg/kg, p.o., 15 days	STZ-induced cognitive impairment in rats	Improved passive avoidance and MWM tests, reduced TBARS, elevated glutathione	(136)
	1-10 μM	Amyloid β1-42-induced murine hippocampal cell death	Attenuated oxidative stress, neuroprotective against cytotoxicity in hippocampal cells	(137)
Safranal	20 mg/kg, p.o., 2 weeks	Diabetic KK-Ay mice	Improved glucose uptake, impaired glucose tolerance in type 2 diabetic KK-Ay mice	(168)
	72.75-291 mg/kg, IP	Anesthetized rats treated with kainic acid	Reduced extracellular glutamate and aspartate concentrations in the hippocampus	(138)
	0.2 ml/kg, IP, 5 days	Scopolamine-induced learning impairment	Inhibition of impaired acquisition/performance activity, reduced latency time	(123)
	72.5 and 145 mg/kg, IP	MCAO-induced transient focal cerebral ischemia in rats	Decreased free radical production, increased antioxidant activity, protected I/R injury	(139)
	0.15-0.5 ml/kg, IP	Forced swimming test in mice	Decreased immobility time, dopamine and norepinephrine uptake, increased swimming climbing time, stereotypic activity	(79)
	0.15 and 0.35 ml/kg, IP	PTZ-induced convulsions in mice	Reduced seizure duration, delay in onset of tonic convulsions, protected mice from death	(87)
	0.025-0.1 mg/kg, IP, 7 days	Allodynia and hyperalgesia-induced CCINP in rats	Alleviated behavioral manifestations of neuropathic pain	(96)
	300 mg/kg, IP, 3 days	Rat model of traumatic injury to the spinal cord	Reduced TNF-α, IL-1β, IL-10 and p38 MAPK, decreased aquaporin-4 expression	(140)
	72-291 mg/kg, IP, 21 days	Quinolinic acid-induced oxidative in rat hippocampus	Protected oxidative markers in the hippocampus	(141)

CCINP: chronic constriction injury model of neuropathic pain, EAE: experimental autoimmune encephalomyelitis, ERK1/2: extracellular signal-regulated kinase1/2, ext.: extract, HSP: hippocampal synaptic plasticity, INPP4: inositol polyphosphate 4-phosphatase, LTP: long‐term potentiation, MCAO: middle cerebral artery occlusion, MHNR: mediated hippocampal neurons responses, MWM: Morris water maze, NMDA: N-methyl-D-aspartate, NPA: nitropropionic acid, NSS: neurological severity score, PDRSM: performance deficits in the recognition and spatial memory, PTZ: pentylenetetrazole, ROS: reactive oxygen species, SCC: spinal cord contusion, SLM: spatial learning and memory, SMD: spatial memory deficit, SOD: superoxide dismutase, SP: synaptic proteins, STZ: streptozotocin, VSMC: vascular smooth muscle cell

**Table 6:** Effects of *Crocus sativus* and its constituents on metabolic disorders in experimental studies

**Compound**	**Dose**	**Study design**	**Possible mechanism and effect**	**Ref.**
Aqueous ext.	40 and 80 mg/kg, IP, 4 weeks	STZ-induced experimental diabetes mellitus	Increased GSH, SOD, and CAT, decreased cognitive deficit, serum TNF-α, induced iNOS activity	(144)
	200 mg/kg, IP, 5 weeks	STZ-induced diabetes in rats	lowered FBG, prevented weight loss	(147)
	100 mg/kg, p.o., 6 weeks	STZ-induced diabetes in rats	Increased hepatocyte structural integrity, reduced diabetic liver injury	(151)
Ethanolic ext.	20-80 mg/kg, p.o., and IP, 14 days	Alloxan-induced diabetes in rats	Decreased insulin immunoreactivity and the number of β-cells in the pancreas	(143)
	40 and 80 mg/kg, p.o., 8 weeks	HFD-fed male Sprague Dawley rats	Improved LDL/HDL ratio, reduced serum TG and TC	(149)
	200-600 mg/kg, p.o., 4 weeks	Alloxan-induced diabetes in rats	Improved pancreas vacuolation of acinar epithelial and Langerhans islets hypertrophy	(152)
	20 mg/kg, p.o.	Hepatic I/R injury	Alleviated ER stress and protein ubiquitination, cell apoptosis, modulated protein oxidation	(153)
	40 mg/kg	STZ-induced hepatic injury in diabetic rats	Hepatoprotective effect, improved antioxidant defense of diabetic liver tissue	(154)
	40 and 80 mg/kg, p.o., 8 weeks	HFD-induced obese rats	Alleviated liver enzymes and histopathological changes	(221)
Methanolic ext.	5-200 mg/kg, p.o., 2 months	Obese Wistar rats	Lowered leptin, reduced fat mass, increased insulin sensitivity	(155)
Aqueous- ethanolic ext.	50 mg/kg, IP, 2 weeks	Healthy male rats	Decreased serum glucose and cholesterol	(142)
	25 and 100 mg/kg, p.o. 21 days	STZ-induced diabetes in rats	Increased adiponectin, regulated glucose and lipid metabolism	(146)
Crocin	5-50 mg/kg, p.o., 2 month	Obese Wistar rats	Lowered leptin, reduced fat mass, increased insulin sensitivity	(155)
	40 and 80 mg/kg, p.o., 8 weeks	HFD-fed male Sprague Dawley rats	Improved LDL/HDL ratio, reduced serum TG and TC	(149)
	50 or 100 mg/kg, IP, 150 days	STZ‐induced diabetes in rats	Decreased serum glucose, TG, TC, and LDL, increased HDL	(156)
	12.5 and 25 mg/kg, IP, 4 weeks	Diazinon-induced hepatotoxicity in rats	Reduced inhibition of ERK activation and hyperlipemia, increased LDL receptor expression	(157)
	12.5-50 mg/kg, IP, 4 weeks	Diazinon-induced hepatotoxicity in rats	Reduced MDA, total caspases-3 and -9, Bax/Bcl-2 ratio, and hepatotoxicity	(158)
	30 and 60 mg/kg, IP, 6 weeks	STZ-induced diabetes in rats	Reduced FBG and lipid peroxidation, increased antioxidant activity	(159)
	10 and 150 mg/kg, p.o., 6 days	Cyclophosphamide‐induced toxicity in rats	Increased glutathione, total thiol, SOD, CAT, GSTs and GPx in liver	(160)
	18.7-75 mg/kg, p.o., 6 weeks	Kunming mice	Enhanced SOD in the liver and kidney, and GSH-Px and TAOC in the liver, heart, and kidney	(161)
Crocetin			Enhanced SOD in the liver and kidney, and GSH-Px and TAOC in the liver, heart, and kidney	(161)
	40 mg/kg, p.o., 2 weeks	Fructose-fed rats	Improved adiponectin, TNF-a, and leptin expression, improved insulin sensitivity	(162)
	2 mg/kg, p.o.	Hemorrhagic shock model	Modified hepatic mRNA expression of cytokines and iNOS in a shock model	(163)
	40 mg/kg, p.o., 6 weeks	Dexamethasone-induced insulin resistance in rats	Prevented insulin resistance development	(164)
	50 mg/kg, IP, 6 weeks	HFD-induced insulin resistance in rats	Inhibited insulin resistance, raised hepatic lipoprotein lipase activity, non‐esterified fatty acid uptake, regulated PPAR-α	(165)
Safranal	0.5 mg/kg	Age-induced liver damage in rats	Protected against oxidative stress, increased antioxidant defenses	(166)
	2 ml/kg, IP, 4 weeks	HFD and STZ-induced DT2 in rats	Decreased serum and pancreas TNF-α and IL-1β, and oxidative stress	(167)
	8 and 12 mM	*Escherichia coli* ATP synthase and cell growth	Inhibited wild-type membrane-bound F1FoATP synthase 100% and αR283D mutant enzyme 50%	(169)
	20 mg/kg, p.o., 2 weeks	Impaired glucose tolerance in DT2 KK‐Ay mice	Improved glucose tolerance, modified catalytic cysteinyl thiol	(168)

CAT: catalase, DT2: type 2 diabetes ext.: extract, FBG: fasting blood glucose, GPx: glutathione peroxidase, GSTs: glutathione‐S‐transferase, HDL: high-density lipoprotein, HFD: high-fat diet, I/R: ischemia‐reperfusion, iNOS: Inducible nitric oxide synthase, LDL: low-density lipoprotein, PAB: prooxidant-antioxidant balance, PCRCT: placebo-controlled, randomized, clinical trial, SOD: superoxide dismutase, STZ: Streptozotocin, TC: total cholesterol, TG: triacylglycerol, PPAR-α: peroxisome proliferator‐activated receptor‐α

**Table 7:** Gastrointestinal effects of *Crocus sativus* and its constituents in experimental studies

**Compound**	**Dose**	**Study design**	**Possible mechanism and effect**	**Ref.**
Aqueous ext.	1%, p.o., 3 days	SDS-induced intestinal damage	Protected against intestinal injury	(170)
Ethanolic ext.	25-250 mg/kg, p.o.	Indomethacin-induced gastric ulcers in diabetic and nondiabetic rats	Prevented gastric mucosa damage, increased glutathione, and diminished lipid peroxidation	(172)
Aqueous- ethanolic ext.	60 mg/ml, IP	Guinea pig ileum stimulated by EFS and epinephrine	Reduced contractile responses of ileum	(171)
*C. Sativus*	90 mg, p.o., 3 days	Hexobarbital-induced in mice	Decreased spontaneous motor activity, prolonged sleeping time, stimulated spontaneous uterus contractility, prevented hemolysis	(179)
Crocin	7.5-30 mg/kg, IP	Indomethacin-induced gastric lesions in rats	Astro-protective effects, reduced MDA, iNOS, and caspase-3, inhibited reduction in mucus content	(173)
	2.5-10 mg/kg, p.o.	Indomethacin-induced gastric ulcers in diabetic and nondiabetic rats	Prevented gastric mucosa damage, increased glutathione, diminished lipid peroxidation	(172)
	18.93 µg/ml	Helicobacter pylori, malarial, leishmanial	Inhibited FabZ and HpPDF	(178)
Crocetin	50-100 mg/kg, IP, 50 days	Human gastric cancer cells and rat model of gastric cancer	Inhibited tumor progression, reversed antioxidant activity and lactate dehydrogenase in serum	(174)
	100 and 200 mg/kg, IP	Burn-induced intestinal injury in rats	Decreased free radicals and lipid peroxidation, neutrophil, TNFα, IL-6, and histological changes, increased antioxidant enzymes	(175)
	50 mg/kg, i.g., 8 days	TNBS-induced colitis in mice	Reduced neutrophil infiltration, lipid peroxidation, and NO, down-regulated NF-κB	(49)
Safranal	0.25-5 mg/kg, p.o.	Indomethacin-induced gastric ulcers in diabetic and nondiabetic rats	Prevented gastric mucosa damage, increased glutathione, diminished lipid peroxidation	(172)
	0.063-1 mg/kg, IP, 7 days	Indomethacin-induced gastric ulcer in rats	Alleviated congestion, inflammatory cell infiltration, edema, gastric mucosa sloughing	(176)
	32 µg/ml	Helicobacter pylori, malarial, leishmanial	Inhibited FabZ and HpPDF	(178)

EFS: electrical field stimulation, ext.: extract, iNOS: inducible nitric oxide synthase, MDA: malondialdehyde, NO: nitric oxide, TNBS: 2,4,6-Trinitrobenzene sulfonic acid

**Table 8:** Respiratory effects of *Crocus sativus* and its constituents in experimental studies

**Compound**	**Dose**	**Study design**	**Possible mechanism and effect**	**Ref.**
*C. Sativus*	0.1-0.4 mg/ml	OVA-sensitized guinea pigs	Reduced serum ET-1 and TP	(183)
Ethanolic ext.	100-800 mg/kg	Citric acid-induced cough in guinea pigs	Reduced coughs	(184)
	0.2 g%	Isoprenaline-induced TSM relaxation	Stimulatory effect on *β*2-adrenoceptors	(185)
Aqueous- ethanolic ext.	20-80 mg/kg	OVA-sensitized guinea pigs	Prevented TR and serum inflammatory mediators, increased Th1/Th2 balance	(180)
	50, 100 and 200 mg/kg	OVA-sensitized rats	Alleviated lung inflammatory cells especially eosinophils in BALF	(181)
	50-200 mg/kg	Experimental asthmatic rats	Decreased WBC count and eosinophil, increased lymphocyte percentage	(181)
	0.025-0.1 mg/ml	Guinea‐pig isolated trachea	Inhibited histamine (H1) receptors	(187)
	0.1-0.4 mg/ml, p.o., 18 days	OVA-sensitized guinea-pigs	Improved lung pathological changes, total and differential WBC, and serum histamine	(182)
			Improved total and differential WBC	(182)
	0.15-0.60 g%	Guinea‐pig isolated trachea	Relaxant effect on TSM	(186)
	4-16 μg/ml, p.o., 18 days	OVA-sensitized guinea pigs	Decreased TR and serum cytokine, total NO, and nitrite, increased Th1/Th2 balance	(200)
	25-100 µg/ml, p,o., 8 days	Guinea‐pig isolated trachea	Competitive antagonistic effect on muscarinic receptors	(199)
Crocin	30-120 μM	Rat isolated trachea	Relaxant effects on TSM	(189)
	100 mg/kg	Murine model of allergic disease	Reduced BALF IL-4, IL-5, IL-13, tryptase, EPX, serum OVA-specific IgE, lung eotaxin, p-ERK, p-JNK, and p-p38 expression	(190)
	25 mg/kg	Murine model of allergic asthma	Reduced inflammatory cytokine expression, restoration of oxidant/antioxidant homeostasis	(191)
	12.5, 25 and 50 mg/kg	Cigarette smoke-induced COPD	Modulated Nrf2 pathway, protected lung injury caused by COPD	(192)
	50 mg/kg	LPS-induced acute lung injury in mice	Reduced NO in lung and iNOS expression	(193)
Crocetin	50 and 100 mg/kg	LPS-induced acute lung injury in mice	Reduced phospho-IκB expression and NF-κB activity in lungs	(194)
Safranal	4-16 µg/ml	OVA-sensitized guinea pigs	Reduced serum ET-1 and TP	(183)
	0.25-0.75 ml/kg	Citric acid-induced cough, guinea pigs	Reduced coughs	(184)
	1.25 and 2.5 μg	Isoprenaline-induced TSM relaxation	Stimulatory effect on *β*2-adrenoceptors	(185)
	4-6 µg/ml, p.o., 18 days	OVA-sensitized guinea pigs	Improved lung pathological changes, total and differential WBC, and serum histamine	(182)
	0.25-0.75 mg/kg	Respiratory distress in diabetic rats	Increased GSH, CAT, and SOD	(201)
	0.1, 0.5 and 1 mM	PHA-stimulated PBMC	Reduced IFN-γ, IL-10, and cell viability, increased IFN-γ and IFN-γ/IL-4 ratio	(202)
	1.25 and 2.5 μg/ml	Guinea‐pig isolated trachea	Adrenoceptor stimulation and muscarinic receptor inhibition	(198)
	4-16 µg/ml, p.o., 18 days	OVA-sensitized guinea pigs	Improved total and differential WBC	(182)
	0.15-0.60 ml	Guinea‐pig isolated trachea	Relaxant effect on TSM	(186)
	0.63-2.5 µg/ml, p,o., 8 days	Guinea‐pig isolated trachea	Competitive antagonistic effect on muscarinic receptors	(199)

BALF: bronchoalveolar lavage fluid, EPX: eosinophil peroxidase, ext.: extract, LPS: lipopolysaccharide, OVA: ovalbumin, PBMC peripheral blood mononuclear cells, PHA: phytohemagglutinin, TR: tracheal responsiveness, TSM: tracheal smooth muscle, COPD: Chronic obstructive pulmonary diseases

**Table 9:** Renal and urogenital effects of *Crocus sativus* and its constituents in experimental studies

**Compound**	**Dose**	**Study design**	**Possible mechanism and effect**	**Ref.**
Aqueous ext.	90 mg/kg, IP	Descriptive study in cats	Diuretic effect, increased glomerular filtration rate	(205)
	5-80 mg/kg	I/R-induced kidney oxidative damage in rats	Reduced lipid peroxidation, increased antioxidants, prevented renal oxidative injury	(210)
	80-320 mg/kg	Normal male rats	Increased MF, IF, and EF behaviors and reduced EL, IL, and ML parameters	(214)
Aqueous- ethanolic ext.	0.5-2 g/kg, p.o.	Acute nephrotoxicity and hepatotoxicity in mice	Decreased serum urea nitrogen and histopathology changes in the kidney	(1)
	40 and 80 mg/kg	Gentamicin-induced nephrotoxicity in rats	Ameliorated harmful effects of GM on kidney	(206)
	10 and 20 mg/kg	Acetaminophen-induced renal damage in rats	Reduced serum creatinine and uric acid, prevented acute nephrotoxicity	(207)
	5, 10, or 20 mg/kg	I/R‐induced acute kidney injury	Decreased serum creatinine, MDAl, TNF-α, leukocyte infiltration, and ICAM-1 expression	(208)
*C. Sativus*	10, 40 and 90 mg/kg	Healthy male rats	Anti-oxidant property, improved epithelial cell function and vascular blood flow	(204)
	100 mg/kg	Spermatogenesis in adult rats	Reversed decreasing free serum testosterone, decreased lipid peroxidation activity	(213)
Crocin	100-400 mg/kg	Normal male rats	Increased MF, IF, and EF behaviors, reduced EL, IL, and ML parameters	(214)
	50-400 mg/kg	I/R-induced kidney oxidative damage in rats	Reduced lipid peroxidation, increased antioxidants, prevented renal oxidative injury	(210)
	20 mg/kg	STZ-induced diabetic nephropathy in rats	Reduced MDA, NO, kidney TLR4 and IL-6, enhanced SOD, GSH, and serum CAT, improved kidney histopathology	(211)
	10 and 20 mg/kg	Cyclophosphamide induced-testicular toxicity	Decreased testicular apoptosis, reduced caspase 3 activity	(216)
Safranal	0.1-0.4 ml/kg	Normal male rats	Increased MF, IF, and EF behaviors, reduced EL, IL, and ML parameters	(214)

CAT: catalase, ext.: extract, I/R: ischemia/reperfusion, ICAM-1: intercellular adhesion molecule‐1, MDA: malondialdehyde, TNF-α: tumor necrotic factor‐alpha

## Conclusion

The findings of multiple experimental studies have demonstrated that *C. sativus* and its primary constituents, including crocin, crocetin, and safranal, possess significant potential in the treatment of a wide spectrum of diseases. This comprehensive review aims to provide an overview of the pharmacological effects of *C. sativus* and its main constituents in both traditional and modern medicine. Notably, the plant and its derivatives have been reported to exhibit preventive or therapeutic effects against cancer, as well as the treatment of various disorders such as cardiovascular, central nervous system, metabolic, gastrointestinal, respiratory, renal, and urogenital disorders. Furthermore, the anti-proliferative, anti-genotoxic, apoptogenic, chemoprotective, and cytotoxic effects of *C. sativus* on various types of cancer have been demonstrated.

In the realm of the cardiovascular system, *C. sativus* and its constituents exhibit an ameliorative impact on cardiac hemodynamic function. Furthermore, they succeed in reducing blood pressure and I/R damage in the ischemic region using modulating beta and alpha receptors, as well as possessing anti-inflammatory and anti-oxidant properties.

The therapeutic effects of *C. sativus* and its components extend to neurodegenerative diseases, such as Alzheimer’s and Parkinson’s. This is achieved through the suppression of pro-inflammatory gene expression and the inhibition of inflammatory mediators in microglia cells.

By acting upon β2 adrenergic, muscarinic, and histamine receptors, the plant and its derivatives induce relaxation in the airways. Additionally, the plant exhibits a preventive effect on inflammatory lung disorders owing to its anti-inflammatory, immunomodulatory, and anti-oxidant properties.

Through the effect on histamine receptors in the stomach, *C. sativus *and its component reduce acid secretion and help to improve the mucosal defense layer. The plant and its derivatives also modulate the effects of menopause and premenstrual syndrome in women and prostate disorders in men, strengthen the anti-oxidant defense system, and reduce the disease process.

A multitude of studies have been conducted to investigate the impact of *C. sativus* and its constituents on various disorders using both *in vivo* and *in vitro* laboratory animal models. While these investigations are essential, they are not sufficient, and further clinical trials are imperative to explore the unknown aspects of the therapeutic effects of *C. sativus* and its primary constituents on diverse disorders.

## Authors’ Contributions

MH B conceived and designed the study. Z G, A M, S B, and MR A performed research. S S and A G wrote the paper. All authors read and approved the manuscript. The authors declare that all data were generated in-house and that no paper mill was used.

## Funding

The authors declare that no funds, grants, or other support were received during the preparation of this manuscript.

## Conflicts of Interest

The authors declare that they have no conflicts of interest.
